# Knockdown of lncRNA MALAT1 attenuates renal interstitial fibrosis through miR-124-3p/ITGB1 axis

**DOI:** 10.1038/s41598-023-45188-y

**Published:** 2023-10-23

**Authors:** Weiping Xia, Xiang Chen, Zewu Zhu, Hequn Chen, Bingsheng Li, Kangning Wang, Li Huang, Zhi Liu, Zhi Chen

**Affiliations:** 1 Department of Urology, Xiangya Hospital, Central South University, Xiangya Road 88, Changsha, 410008 Hunan People’s Republic of China; 2 National Clinical Research Center for Geriatric Disorders, Xiangya Hospital, Central South University, Changsha, China; 3 Department of Intensive Care Medicine, Xiangya Hospital, Central South University, Changsha, 410008 Hunan People’s Republic of China

**Keywords:** Genetics, Urology

## Abstract

Renal interstitial fibrosis (RIF) considered the primary irreversible cause of chronic kidney disease. Recently, accumulating studies demonstrated that lncRNAs play an important role in the pathogenesis of RIF. However, the underlying exact mechanism of lncRNA MALAT1 in RIF remains barely known. Here, the aim of our study was to investigate the dysregulate expression of lncRNA MALAT1 in TGF-β1 treated HK2/NRK-49F cells and unilateral ureteral obstruction (UUO) mice model, defining its effects on HK2/NRK-49F cells and UUO mice fibrosis process through the miR-124-3p/ITGB1 signaling axis. It was found that lncRNA MALAT1 and ITGB1 was significantly overexpression, while miR-124-3p was downregulated in HK2/NRK-49F cells induced by TGF-β1 and in UUO mice model. Moreover, knockdown of lncRNA MALAT1 remarkably downregulated the proteins level of fibrosis-related markers, ITGB1, and upregulated the expression of epithelial marker E-cadherin. Consistently, mechanistic studies showed that miR-124-3p can directly binds to lncRNA MALAT1 and ITGB1. And the protect effect of Len-sh-MALAT1 on fibrosis related protein levels could be partially reversed by co-transfected with inhibitor-miR-124-3p. Moreover, the expression trend of LncRNA MALAT1/miR-124-3p/ITGB1 in renal tissues of patients with obstructive nephropathy (ON) was consistent with the results of cell and animal experiments. Taken together, these results indicated that lncRNA MALAT1 could promote RIF process in vitro and in vivo via the miR-124-3p/ITGB1 signaling pathway. These findings suggest a new regulatory pathway involving lncRNA MALAT1, which probably serves as a potential therapeutic target for RIF.

## Introduction

Chronic kidney disease (CKD), which affect the health of more than 10% of general population^1^, is one of the most risk factors of end-stage kidney disease (ESKD) worldwide^2,3^. Renal interstitial fibrosis (RIF) is usually considered the ultimate common pathway for almost all kidney diseases leading to ESKD^4,5^. RIF, a wound‐healing response^6^, is characterized by epithelial-to-mesenchymal transition (EMT) in tubular epithelial cells (TECs)^7^, massive fibroblast activation, and excessive extracellular matrix (ECM) deposition^8^, leading to renal scar tissue formation, thereby promoting the incidence of ESKD. As we all known, continuous injury of tubular epithelial cells can promote dysregulation of key RIF processes, such as epithelial dedifferentiation, cell cycle arrest, and profibrotic factors secretion activate myofibroblasts differentiation and proliferation^9–11^. Therefore, the acquisition of mesenchymal phenotype after epithelial cell injury plays an important role in the process of RIF. Although many studies have been put into finding the molecular mechanisms and cellular regulators of RIF in recent years, there are currently no suitable therapies to prevent the occurrence or progression of RIF^12^. In addition, TGF‐β1, a pro‐fibrotic cytokine, which is the most important inducer in fibrosis diseases^13^, especially in RIF^14^. However, the exact molecular mechanisms by which epithelial cells acquired mesenchymal phenotype and renal interstitial fibroblasts activation in RIF is still not fully understood by us. Thus, inhibition of the progression of RIF induced by TGF-β1 might be a crucial treatment for preventing the progression of CKD.

Long non-coding RNAs (lncRNAs) are defined as classes of endogenous non coding transcripts with a length of over 200 nucleotides^15,16^, which controlling fundamental cellular processes at multiple levels, such as protein-coding gene expression at the epigenetic, transcriptional, and post-transcriptional levels^17,18^. As a hot topic in research, large number of studies have demonstrated that lncRNAs can act as competing endogenous RNA (adsorbed miRNA) and then abolishing their suppression of mRNA translation^19,20^. Recently, lncRNA metastasis associated lung adenocarcinoma transcript 1(MALAT1) has received attention from investigators due to its important role in in different type of cancers^21^. Simultaneously, prior studies have reported that lncRNA MALAT1 were involved in fibrosis in various organs, including the heart, liver, lung and gain more attention^22–25^. However, little is known of the role of lncRNA MALAT1 in the development or progression of RIF. Huang et al. reported that lncRNA MALAT1 upregulation significantly facilitated the expression of collagen I, collagen IV, fibronectin (FN) under high glucose stimulated HK-2 cells and model of streptozotocin-induced diabetic rats^26^. Liu et al.^27^ proved that lncRNA MALAT1 was increased in patients with obstructive nephropathy (ON) and TGF-β1 induced HK2 cells, and might involve in RIF process by m6A modification. Hence, further studies to identify and characterize the specific mechanisms of lncRNA MALAT1 in RIF progression are critical for the development of new therapies to delay or reverse RIF disease.

To our knowledge, miR-124-3p, which is emerging as a potent regulator of gene expression and disease pathogenesis, is one of common studied miRNA in fibrosis disease. The cardiac fibroblasts specific ceRNA network including miR-124-3p, is crucial to cardiac fibrosis^28^. Jin et al.^29,30^ demonstrated that miR-124-3p was a downstream effector to be involved in HOXA11-AS-mediated phenotypes through directly targeting TGFβR1 or Smad5 signaling, contributing to the progression of keloid formation. Chen et al.^31^ revealed that porcine acellular dermal matrix promoted wound healing and scar formation through inhibited the expression of miR-124-3p.1. However, the role of miR-124-3p in RIF is still unknown.

In addition, ITGB1, an important ECM receptor, is the most abundantly expressed β-integrin subunit in the kidney^32^. ITGB1 is a critical factor in the regulation of renal structure and function^33^. Recently, it is reported that inhibition of ITGB1 expression counteracted TGF-β1 induced EMT process in renal TECs and UUO mice model^34^.

Therefore, this study was conducted to better understand whether the MALAT1/miR-124-3p/ITGB1 signaling axis is involved in mediating the biological functions in vitro and in vivo model of RIF.

## Material and methods

### Clinical sample collection

The relatively normal renal tissue specimens of 20 patients who underwent radical nephrectomy due to renal tumors (tumors confined to the upper or lower pole of the kidney) and trauma in Xiangya Hospital of Central South University were collected as a control group. Furthermore, twenty patients with severe hydronephrosis due to obstruction requiring radical nephrectomy in Xiangya Hospital of Central South University were enrolled as experimental group. Studies using human tissues were approved by the Human Research Ethics Committees of Xiangya Hospital of Central South University (2,022,100,947), and all patients provided written informed consent. This study followed the Declaration of Helsinki, internationally recognized ethical standards, and relevant Chinese laws and regulations. All collected samples were eligible for experimental use. These specimens were obtained for further experiments.

### Animal model

Animals were handled according to a protocol approved by the Laboratory Animal Care of Central South University of China (No.2021sydw0088). All animal experiments in this study were conducted in accordance with the ARRIVE guidelines for reporting experiments involving animals^35^. And all methods were carried out in accordance with the relevant guidelines and regulations.

Twenty-eight specific‐pathogen‐free (SPF) male and healthy C57BL/6 J mice (aged 9 weeks; 20–22 g body weight) provided by Hunan SLAC Laboratory Animal Co., Ltd. (Hunan, China) were selected for this experiment. All mice were housed under conditions of temperature at 25 °C, relative humidity (60%), and a constant 12 h light–dark periods daily with free access to standard food and water. After one week of acclimatization to the feeding environment, a total of 28 mice were randomly divided into the following four groups (n = 7 per group): sham operation group (sham), UUO groups, UUO + Len-sh-control group and UUO + Len-sh-MALAT1 group. To be specific, mice were fasted for 1 h at least before the experiments and then were anesthetized by the intraperitoneal injection of 1% pentobarbital sodium. After that, the posterior abdominal cavity was opened in lateral decubitus position. In the sham operation groups, only the left ureter was separated without ligation and then the abdominal incision was closed. The UUO groups just under the same surgery condition but ligation the left ureter by using suture materials (4–0 silk). Short hairpin RNA targeting MALAT1 (Len-sh-MALAT1), along with the scramble control lentiviruses (Len-sh-control) were generated by GenePharma (Shanghai, China). 100 μL containing of 2 × 10^7^ TU Len-sh-MALAT1/Len-sh-control in PBS was injected into mice via tail vein injection as UUO + Len-sh-MALAT1/UUO + Len-sh-control mice model^36,37^. All surgical procedures were performed in a sterile environment. Because some mice died after anesthesia accident, surgical intervention and lentivirus injection during the experiment, 4 mice in each group were reserved for subsequent statistical analysis. The mice were sacrificed via exposure in a carbon dioxide environment after 2 weeks, and the kidney on the ligated side were collected for further experiments.

### Cell culture and reagents

The human proximal tubular cell line (HK-2), NRK-49F, HEK-293 T cells were obtained from the Procell Life Science and Technology (Wuhan, China). Cells were incubated in Dulbecco’s modified Eagle’s medium (BI, Israel) supplemented with 10% fetal bovine serum (BI, Israel), 0.1 mg/mL streptomycin (BI, Israel), and 100 U/mL penicillin (BI, Israel) in an incubator containing 5% CO2 at 37 °C. HK-2, NRK-49F cells were treated with TGF-β1 (0, 2.5, 5, 10 ng/mL) for 24 h to construct a cell fibrosis model, and the extracted RNA or protein was carried out for further experiment. TGF-β1 was purchased from Sino Biological (Beijing, China, No.10804-HNAC) and dissolved in dimethyl sulfoxide.

### Cell transfection

The lentiviruses carrying Len-sh-MALAT1 and corresponding scramble control lentiviruses (Len-sh-control) were obtained from GenePharma (Shanghai, China). Mimic-miR-124-3p, inhibitor-miR-124-3p, mimic-mir-124-3p, inhibitor-mir-124-3p and negative controls (mimic NC/inhibitor NC) were purchased from Sangon Biotech (Shanghai, China).

Before lentiviruses intervention, cells were seeded in the 6-well plate, and the serum free medium was discarded 12 h after transfection (MOI = 5) and replaced with fresh medium contain 10% fetal bovine serum. The infected HK2/NRK-49F cells were then screened with 2 mg/mL puromycin for at least 5 days.

To regulate the expression of miR-124-3p in the two cell lines, cells at 70% confluence were transfected with inhibitor NC (150 nmol/L), inhibitor-miR-124-3p (150 nmol/L), mimic NC (150 nmol/L), mimic-miR-124-3p (150 nmol/L) by using Lipofectamine 2000 (Invitrogen, United States) according to the manufacturer’s protocols. After that, the cells were used in the subsequent experiments.

### RNA extraction and quantitative real-time polymerase chain reaction (qRT-PCR)

Total RNA was prepared from clinical samples, mice kidney tissues or cultured HK2/NRK-49F cells using TRIzol reagent (Takara, Japan) and then reverse transcription PCR was performed using a PrimeScript RT reagent Kit (Takara, Japan) for lncRNA MALAT1 and ITGB1. miRNA reverse transcription kit (Accurate Biotechnology, China) for miR-124-3p. qRT-PCR was conducted using SYBR Green PCR reagent (Takara, Japan) in a real-time PCR system (Applied Biosystems, United States). The mRNA expression levels of lncRNA MALAT1, ITGB1, and miR-124-3p were normalized to GAPDH or U6, and analyzed by 2^−ΔΔCt^ method. Furthermore, The PCR primers were obtained from Sangon Biotech (Shanghai, China), and the PCR primer sequences information were shown in Supplementary Table 1.

### Western blot (WB)

Total protein was prepared from clinical samples, mice kidney tissues or cultured HK2/NRK-49F cells using RIPA lysis buffer (NCM, China) mixed with 1% PMSF (NCM, China), And the BCA Protein Assay Kit (Beyotime Biotechnology, China) was used to quantified the concentration of protein. Then the same amounts of protein were separated by 10% sodium dodecyl sulfate polyacrylamide gel electrophoresis (SDS-PAGE) (EpiZyme, China) and were transferred onto polyvinylidene fluoride (PVDF) membrane (Merck Millipore, Germany) soon after. The membranes were blocked by protein free quick blocking solution (EpiZyme, China, Lot:034C1300) for 10 min at room temperature and incubated overnight with primary antibody overnight at 4 °C. The details of the primary antibodies are included anti-glyceraldehyde 3-phosphate dehydrogenase (anti-GAPDH 1:4000, ab8245, Abcam, United Kingdom), anti-E-cadherin (1:2000, ab76055, Abcam, United Kingdom), anti-N-cadherin (1:1000, sc-59987, Santa), anti-vimentin (1:1000, ab92547, Abcam, United Kingdom), anti-α-SMA (ab124964, 1:3000, Abcam, United Kingdom), anti-integrin β1 (1:2000, ab179471, United Kingdom), anti-collagen 1 (1:1000, 14,695–1-AP, Proteintech), anti-fibronectin (1:1000, 66,042–1-Ig, Proteintech), anti-MMP2 (1:1000, 66,366–1-Ig, Proteintech), anti-MMP9 (1:1000, 103,752-AP, Proteintech). After this, the membranes were washings three times with TBST for 10 min each and incubated with the secondary antibody for 1 h at room temperature by using the goat anti-rabbit or goat anti-mouse IgG (1:5000; Proteintech, China). Finally, the enhanced chemiluminescence (ECL) detection kit (NCM Biotech; China) was used to detected the protein blot band. The band was use GAPDH as the internal reference. Furthermore, the band gray value was measured by quantity one software (Bio-Rad, Berkeley, CA, United States). The relative densities of the protein bands were calculated with ImageJ software. Due to the close molecular weight of some of the target genes, our original bands appear to be closely cropped. Original blot images are provided in supplementary raw materials.

### Scratch assay

In order to explore miR-124-3p is regulated the process of EMT and fibrosis in HK-2, NRK-49F cells. Cells were seeded in the 6-well plate, cells at 60% confluence were transfected with inhibitor NC (150 nmol/L), inhibitor-miR-124-3p (150 nmol/L) by using Lipofectamine 2000 (Invitrogen, United States) according to the manufacturer’s protocols. After 48 h, TGF-β1 (10 ng/mL) was added to TGF-β1 group, TGF-β1 + inhibitor NC group, TGF-β1 + inhibitor-miR-124-3p group, respectively, and the scratch assay was made using a sterile 200ul pipette tip. The cells were then incubated with FBS-free culture medium alone. Images of the scratches were captured at 0 and 24 h with light microscopy at 50× magnification. The width of the scratch was analyzed using ImageJ software.

### Cell counting Kit-8 assay

Cell proliferation were estimated by the Cell Counting Kit-8 (CCK-8) assay (NCM Biotech; China). Cells were seeded in the 6-well plate, cells at 60% confluence were transfected with inhibitor NC (150 nmol/L), inhibitor-miR-124-3p (150 nmol/L) by using Lipofectamine 2000 (Invitrogen, United States) according to the manufacturer’s protocols. After 48 h, cells were trypsinized and dispensed into individual wells of 96-well plates with a density of 3000 cells per well. And then TGF-β1 group, TGF-β1 + inhibitor NC group and TGF-β1 + inhibitor mir-124-3p group were treated with TGF-β1 (10 ng/mL) after cell adhesion, respectively. After 24 h cultivation, each well was incubated with CCK-8 solution for 2 h away from light before measuring the absorbance at 450 nm by BioTek Epoch.

### Dual-luciferase reporter assays

Bioinformatics analysis showed that lncRNA MALAT1 or ITGB1 might contain putative binding sites of miR-124-3p, dual luciferase reporter assays were implemented to exam the direct interactions between lncRNA MALAT1 and miR-124-3p as well as miR-124-3p and the 3′-untranslated region (3′-UTR) of ITGB1 mRNA. The sequences of lncRNA MALAT1 and 3′-UTR of ITGB1 including wild-type (WT) binding sites or mutant binding (MUT) sites were amplified and inserted into pSI-Check2 vector (HanBio, shanghai, China), respectively. The HEK-293 T cells were co-transfected with miR-124-3p mimics + lncRNA MALAT1-WT or lncRNA MALAT1-Mut, mimics-NC + lncRNA MALAT1-WT or lncRNA MALAT1-Mut, co-transfected with miR-124-3p mimics + ITGB1-WT or ITGB1-Mut, mimics-NC + ITGB1-WT or ITGB1-Mut for 48 h by using Lipofectamine 2000 reagent (Invitrogen, United States) according to the manufacturer’s protocols. Afterwards, the luciferase activities of renilla and firefly were detected using a dual-luciferase reporter assay system (Vazyme, China).

### Immunohistochemistry (IHC)

The clinical samples and mice kidney tissues were fixed with 4% paraformaldehyde for 48 h, embedded in paraffin, and 4 μm thick sections were prepared. Then, the sections were successively deparaffinized and hydration, antigen retrieval under 95 °C for 15 min, immersed in 10% hydrogen peroxide for 10 min. After this, sections were blocked with 10% normal goat serum at room temperature for 30 min and incubated with primary antibodies using anti-E-cadherin (1:1000, ab76055, Abcam, United Kingdom), anti-N-cadherin (1:200, sc-59987, Santa), anti-α-SMA (1:1000, Abcam, United Kingdom), anti-integrin β1 (1:1000, ab179471, Abcam), anti-collagen 1 (1:300, 14,695–1-AP, Proteintech), anti-fibronectin (1:200, 66,042–1-Ig, Proteintech) overnight at 4 °C. sections were washings three times with PBS for 5 min each and incubated with the biotinylated secondary antibody (OriGene, United States) at room temperature for 50 min, subsequently reaction with DAB chromogen (OriGene, United States), counterstained with hematoxylin (Solarbio, China), and images were acquired with a contrast light microscope (Leica, Germany). Afterward, the positive area was assessed by the ImageJ image analysis software system.

### Hematoxylin and eosin (HE) staining and Masson staining

The clinical samples and mice kidney tissues were fixed with 4% paraformaldehyde for 48 h, embedded in paraffin, and then 4 μm thick sections were prepared. All the sections were then stained with HE staining and Masson trichrome using conventional methods as previously described^38–40^. Images were then obtained under light microscopy at a magnification of 20× (Leica, Germany). The positive area of Masson staining was assessed by the ImageJ image analysis software system.

### Fluorescence in situ hybridization (FISH)

The subcellular localization of lncRNA MALAT1 in HK2 and NRK-49F cells was identified using FISH method. Cy3-labeled probes against 18S rRNA, U6 snRNA, lncRNA MALAT1 (red) were designed and purchased from Servicebio Company (Wuhan, China). In short, HK2 and NRK-49F cells were inoculated into the slides until cell confluence reached about 70%. The glass slides were rinsed with PBS and fixed with 4% paraformaldehyde for 15 min at room temperature. After that, the slides were treated with proteinase K and incubate at 37 °C for 5 min, stringency washed with PBS, and incubated in prehybridization buffer at 37 °C for 1 h and then discarded. Next, hybridization solution containing the lncRNA MALAT1 probe was added to each slide at 42 °C overnight. Subsequently, the slides were rinse with 2 × standard saline citrate (SSC) solution at 37 °C for 5 min, 1 × SSC two times for 5 min each at 37 °C, and wash in 0.5 × SSC at 37 °C for 10 min. the nucleus was stained with 4′,6‐diamidino‐2‐phenylindole (DAPI) for 8 min in the dark. Finally, the slides were sealed with anti-fluorescent quenching agent. Five visual fields were selected to observation and photography under fluorescence microscope (Leica, Germany).

### RNA immunoprecipitation (RIP) assay

A Magna RIP Kit (17–701; Millipore, United States) was applied to determine the interaction between lncRNA MALAT1 and miR-124-3p according to the manufacturer’s instructions. Briefly, HK2 and NRK-49F cells were lysed by using RIP lysis buffer solution. And antibody, including the anti-Ago2 antibody (cs204386; Millipore, United States), normal immunoglobulin G (IgG; included in the kit: 17–701) were used for RIP. The efficiency of Ago2 immunoprecipitation and immunoprecipitated RNA/total RNA (input control) was evaluated by WB and qRT-PCR, respectively.

### Statistical analysis

All experiments were repeated at least three times individually and the data were presented as the mean ± standard deviation (SD). The differences between the two groups were analyzed by independent sample t test; one-way ANOVA was used to determine differences among three or more groups. A two tailed *p* value < 0.05 was considered statistically significant. GraphPad Prism 8 software (GraphPad Software, La Jolla, CA, United States) was used to statistical analyses.

## Results

### lncRNA MALAT1 and ITGB1 are upregulated, while miR-124-3p was downregulated in TGF-β1 induced HK2 and NRK-49F cells

**Figure 1:**
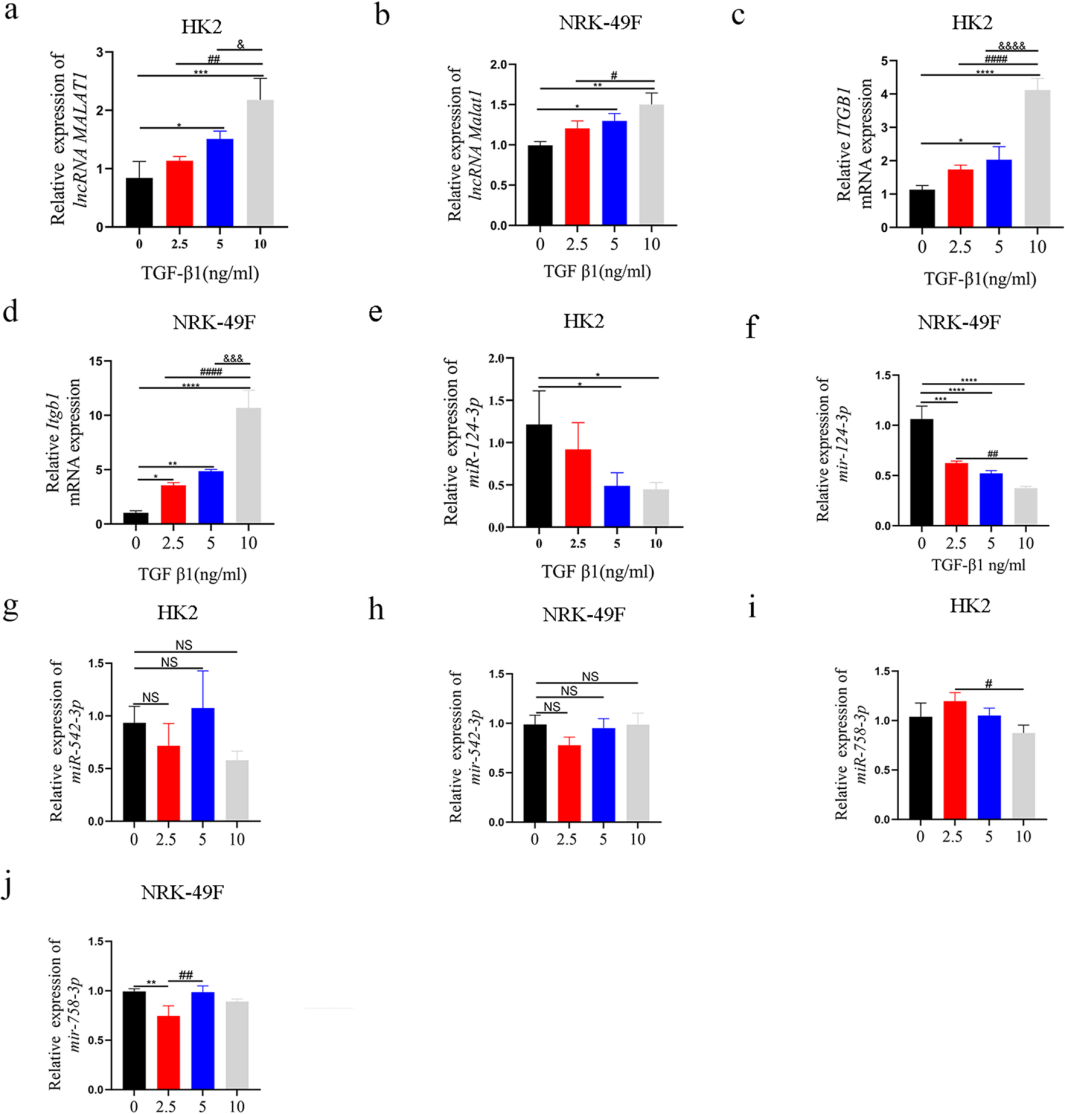
(**a**, **c**, **e**) The qRT-PCR showed that lncRNA MALAT1and ITGB1 were upregulated with the increase of TGF-β1 concentration in HK2 cells, while miR-124-3p was downregulated with the increase of TGF-β1 concentration. (**b**, **d**, **f**) qRT-PCR showed that lncRNA Malat1and Itgb1 were upregulated with the increase of TGF-β1 concentration in NRK-49F cells, while mir-124-3p was downregulated with the increase of TGF-β1 concentration. (**g**, **h**, **i**, **j**) qRT-PCR was used to evaluate the expression of miR-542-3p and miR-758-3p with the increase of TGF-β1 concentration in HK2 and NRK-49F cells. NS = no significance; *: *p* < 0.05;**: *p* < 0.01;***: *p* < 0.001; ****: *p* < 0.0001 (compare with 0 ng/mL); #: *p* < 0.05; ##: *p* < 0.01; ####: *p* < 0.0001(compare with 2.5 ng/mL); &: *p* < 0.05; &&&: *p* < 0.001; &&&&: *p* < 0.0001 (compare with 5 ng/mL).

Initially, we detected the expression levels of lncRNA MALAT1 and ITGB1 in HK2 and NRK-49F cells after TGF-β1 treatment. The results show that lncRNA MALAT1 and ITGB1 RNA levels were up-regulated in HK2 or NRK-49F cells induced by TGF-β1 (Fig. 1a–d). We then used starbase V3.0 for bioinformatics analysis and found that miR-124-3p, miR-542-3p and miR-758-3p predicted to be a potential functional target to interact with lncRNA MALAT1. However, except for miR-124-3p, the expression of miR-542-3p and miR-758-3p was no significant down-regulated in a concentration-dependent manner. (Fig. 1e–j). Hence, the concentration of 10 ng/mL was selected for further experiments. As demonstrated by WB, ITGB1 protein level, EMT process induced by TGF-β1 in HK2 and mesenchymal markers expression in TGF-β1 induced NRK-49F cells were prominently increased (Supplementary Fig. 2a, b). These above results revealed that the abnormal expression of lncRNA MALAT1, miR-124-3p and ITGB1 might imply its important role in RIF.

### Knockdown of lncRNA MALAT1 inhibited EMT process in TGF-β1 induced HK2 and fibrosis-related markers in TGF-β1 induced NRK-49F cells

**Figure 2:**
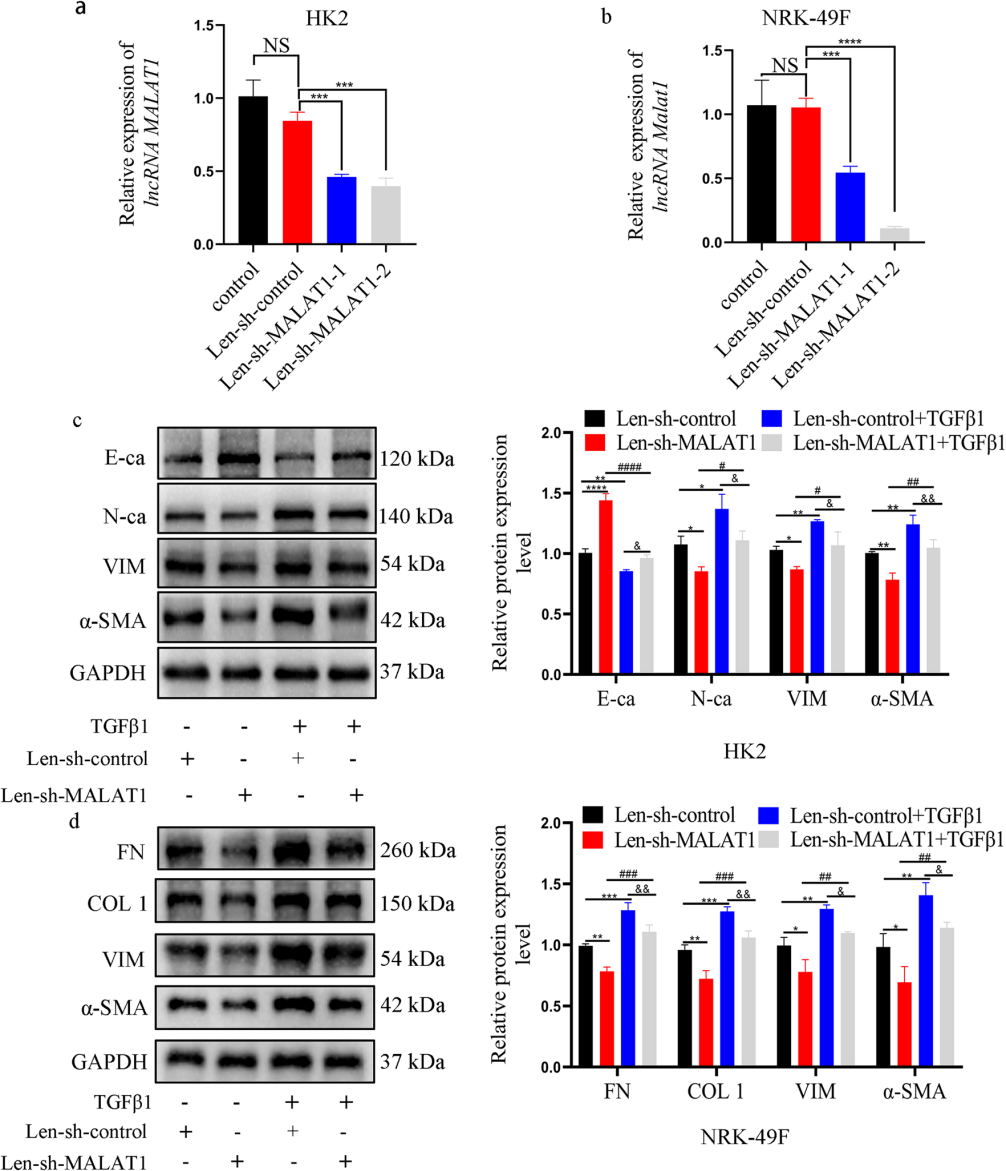
(**a**) The transfection efficiency of knockdown lncRNA MALAT1 was detected by qRT-PCR in HK2 cells. NS = no significance; ***: *p* < 0.001. (**b**) The transfection efficiency of knockdown lncRNA Malat1 was detected by qRT-PCR in NRK-49F cells. NS = no significance; ***: *p* < 0.001. (**c**) After knockdown of lncRNA MALAT1 expression in TGF-β1 induced HK2 cells, WB showed that the expression changes of fibrosis-related markers (E-ca, N-ca VIM, α-SMA). Each assay was implemented in triplicate (*n* = 3). (**d**) After knockdown of lncRNA Malat1 expression in TGF-β1 induced NRK-49F cells, WB showed that the expression changes of fibrosis-related markers (FN, COL1, VIM, α-SMA). Each assay was implemented in triplicate (*n* = 3). *: *p* < 0.05; **: *p* < 0.01; ***: *p* < 0.001; ****: *p* < 0.0001 (Len-sh-MALAT1group and Len-sh-control + TGF β1 group compare with Len-sh-control); #: *p* < 0.05; ##: *p* < 0.01; ###: *p* < 0.001; ####: *p* < 0.0001 (Len-sh-MALAT1 group was compared with Len-sh-MALAT1 + TGF β1group); &: *p* < 0.05; &&: *p* < 0.01 (Len-sh-control + TGF β1 group was compared with Len-sh-MALAT1 + TGF β1 group).

To explore the biological functions of lncRNA MALAT1 in vitro, silencing of lncRNA MALAT1 expression performed by transfected lentiviruses into HK2 and NRK-49F cells. And the results showed that lncRNA MALAT1 expression was significantly downregulated in HK2 and NRK-49F cells transfected with Len-sh-MALAT1-1 and Len-sh-MALAT1-2 by qRT-PCR (Fig. 2a, b). Thus, Len-sh-MALAT1-2 was selected for further experiments. For functional significance, we focused on EMT process and ECM accumulation, two biological phenotypes are important for RIF process. WB results revealed that the proteins level of N-cadherin (N-ca), Vimentin (VIM), and α-SMA were upregulated and the expression of E-cadherin (E-ca) was downregulated in HK2 cells treated by TGF-β1. Furthermore, the proteins level of FN, Collagen1 (COL1), VIM and α-SMA were also upregulated in TGF-β1 induced NRK-49F cells. However, the expression trend of EMT markers and ECM accumulation was significantly counteracted by MALAT1 depletion when comparing with the Len-sh-control + TGF-β1 group (Fig. 2c, d).

### lncRNA MALAT1 is a direct target of miR‐124‐3p

**Figure 3:**
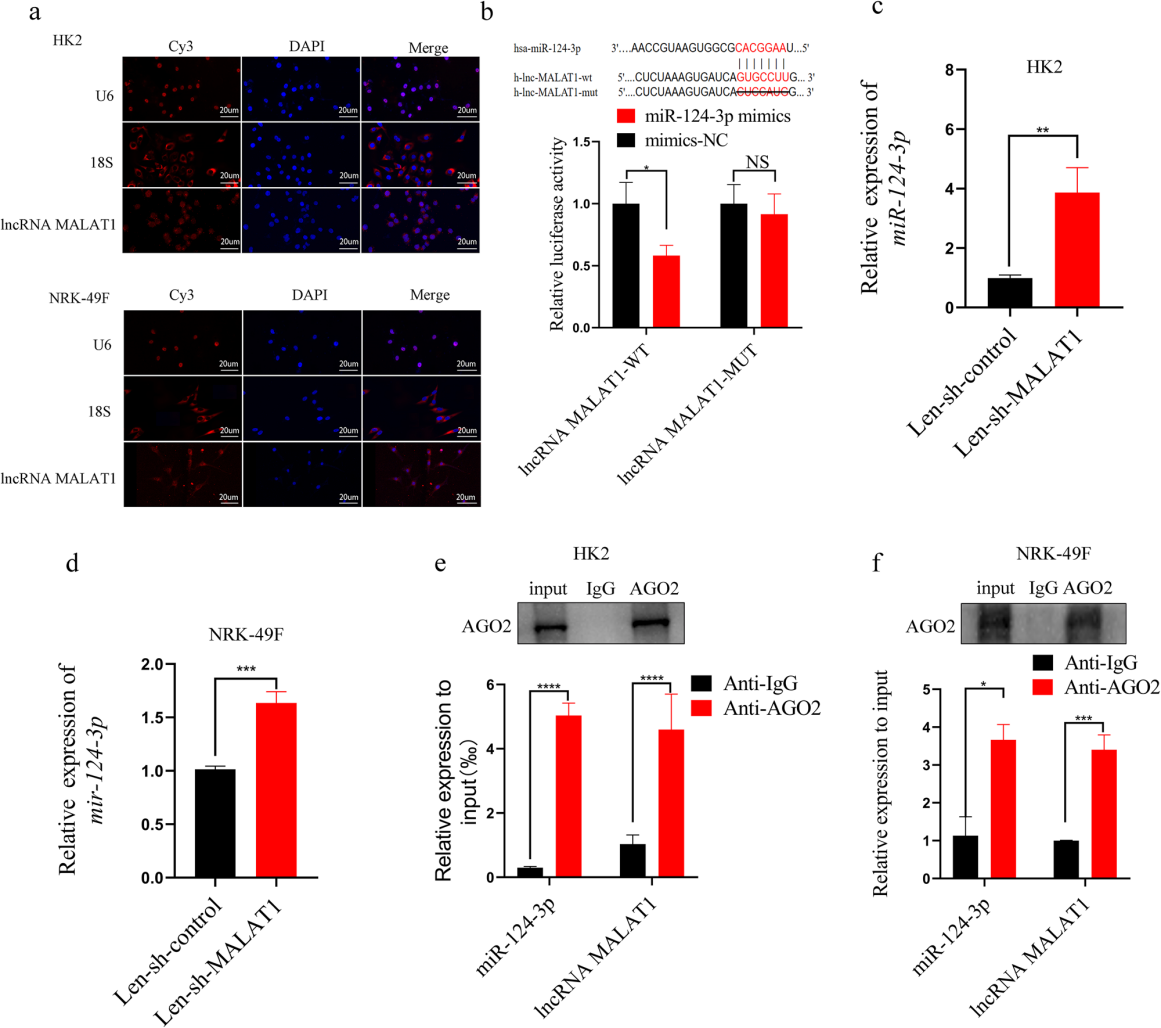
(**a**) The localization of lncRNA MALAT1 in HK2 and NRK-49F cells was detected by FISH assay (bar = 20um)). (**b**) Dual luciferase reporter gene assay verified that lncRNA MALAT1 could directly target miR-124-3p in 293 T cells. (**c**, **d**) qRT-PCR was used to detect the expression of miR-124-3p after transfection with Len-sh-MALAT1 in HK2 and NRK-49F cells. (**e**, **f**) RIP assay was used to detect the direct binding of lncRNA MALAT1 with miR-124-3p in HK2 and NRK-49F cells. NS = no significance; *: *p* < 0.05; **: *p* < 0.01; ***: *p* < 0.001; ****: *p* < 0.0001.

To further illustrate the underlying mechanism by which lncRNA MALAT1 regulated TGF-β1 treated fibrosis process in the two cell lines. We first used FISH method to confirm the localization of lncRNA MALAT1, illustrating that lncRNA MALAT1 was expressed in both the cytoplasm and nucleus of HK2 and NRK-49F cells (Fig. 3a). Next, the result of luciferase reporter assay verified that miR-124-3p overexpression reduced the relative luciferase activity in lncRNA MALAT1 WT group, but no significant difference in lncRNA MALAT1 MUT group in 293 T cells (Fig. 3b). Furthermore, knockdown of lncRNA MALAT1 markedly increased miR-124-3p expression in the two cell lines (Fig. 3c, d). In addition, RIP assay was also performed to verify that whether there is a correlation between miR-124-3p and lncRNA MALAT1. The results showed that the immunoprecipitation of lncRNA MALAT1 and miR-124-3p in Ago2 was significantly higher than that of IgG immunoprecipitation in the two cell lines (Fig. 3e, f). These results manifested that lncRNA MALAT1 and miR-124-3p have a negative regulatory mechanism in HK2 and NRK-49F cells.

### miR‐124‐3p regulated TGF-β1 caused HK2 cells EMT process and fibrosis via targeting ITGB1

**Figure 4:**
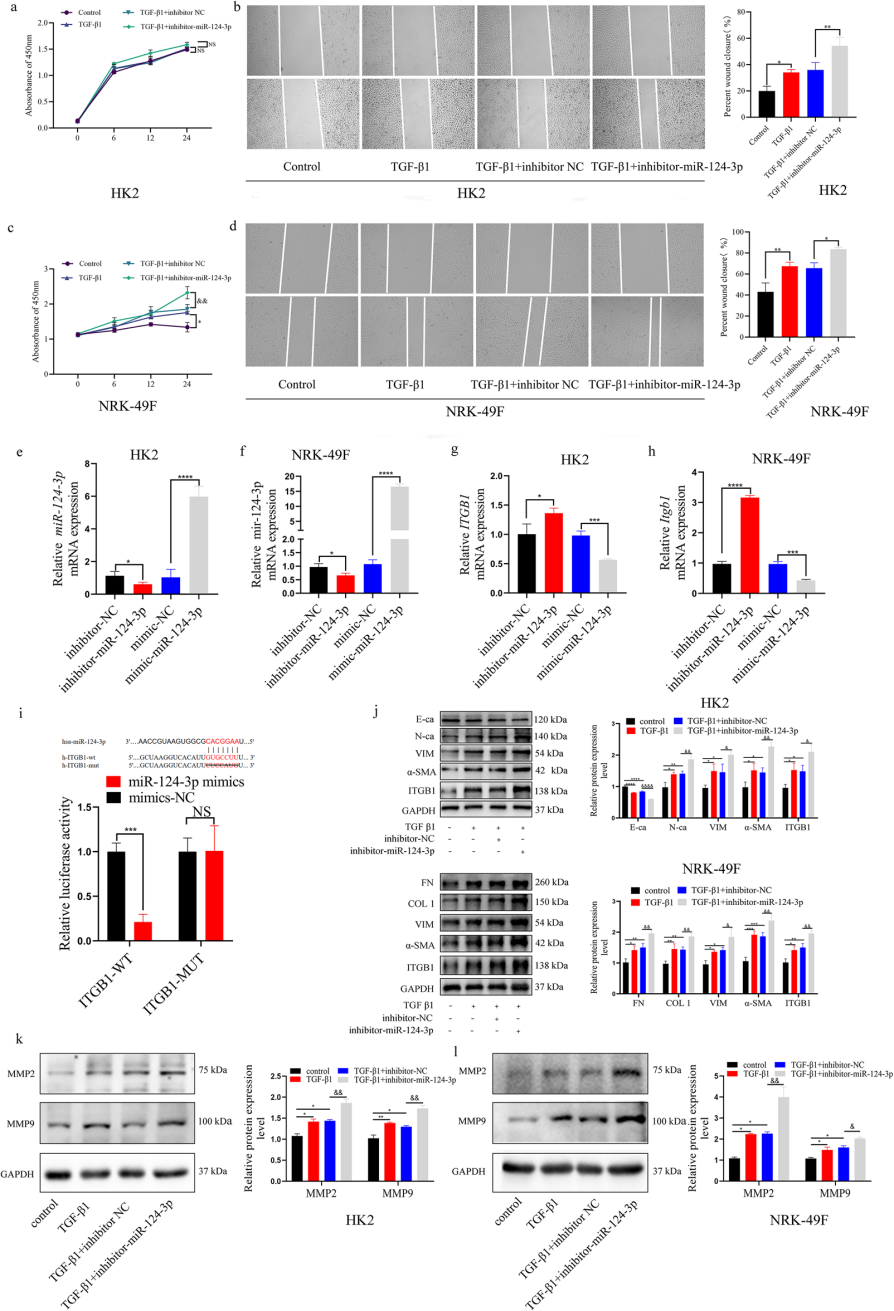
(**a**, **c**) CCK8 was performed to evaluate the proliferative changes after transfection with inhibitor-miR-124-3p in TGF-β1 induced HK2 and NRK-49F cells (from 3 experiments). *: *p* < 0.05 (TGF-β1 group compare with control group). &&: *p* < 0.01 (TGF β1 + inhibitor-miR-124-3p group compare with TGF β1 + inhibitor-NC group). (**b**, **d**) Scratch test was used to evaluate the migration after transfection with inhibitor-miR-124-3p in TGF-β1 induced HK2 and NRK-49F cells (from 3 experiments). *: *p* < 0.05; **: *p* < 0.01. (**e**, **f**, **g**, **h**) qRT-PCR was used to evaluate the expression of miR-124-3p or ITGB1 after transfection with inhibitor-miR-124-3p or mimic-miR-124-3p in HK2 and NRK-49F cells. *: *p* < 0.05; ***: *p* < 0.001; ****: *p* < 0.0001. (**i**) Dual luciferase reporter gene assay confirmed that ITGB1 could directly bind to miR-124-3p in 293 T cells. ***: *p* < 0.001; NS = no significance. (**j**, **k**, **l**) After miR-124-3p was knockdown in TGF-β1 induced HK2 and NRK-49F cells, the expression changes of fibrosis-related markers were detected by WB (from 3 experiments). *: *p* < 0.05; **: *p* < 0.01; ***: *p* < 0.001; ****: *p* < 0.0001 (TGF β1group and TGF β1 + inhibitor-NC group was compare with control group); &: *p* < 0.05; &&: *p* < 0.01; &&&&: *p* < 0.0001 (TGF β1 + inhibitor-NC group was compare with TGF β1 + inhibitor-miR-124-3p group).

To display the underlying mechanism of miR-124-3p, Firstly, results of CCK8 assay showed that the proliferation capacity of HK2 cells induced by TGF-β1 was no change after inhibiting the expression of miR-124-3p (Fig. 4a), while the results of scratch test revealed that cell migration capacities were enhanced (Fig. 4b). In addition, down-regulation of miR-124-3p significantly increased the proliferation, migration ability of NRK-49F cells induced by TGF-β1 (Fig. 4c, d). Moreover, we found that the 3′-untranslated region (UTR) of ITGB1 contained a potential miR-124-3p binding site, which predicted by TargetscanHuman7.1, and the qRT-PCR results showed that miR-124-3p expression was significantly up/downregulated in HK2 or NRK49F cells after transfected with mimic-miR-124-3p or inhibitor-miR-124-3p, respectively (Fig. 4e, f). As shown in Fig. 4g, h, ITGB1 mRNA expression was significantly increased/decreased by transfecting inhibitor-miR-124-3p or mimic-miR-124-3p. After ITGB1-WT luciferase reporting gene plasmids and miR-124-3p mimic were co-transfected into 293 T cells, the luciferase activities were significantly reduced compared with ITGB1-MUT and miR-124 mimic treatment, suggesting that ITGB1 is a direct target of miR‐124‐3p (Fig. 4i). Consistently, the protein level of ITGB1 was up-regulated in the two cell lines treated by TGF-β1, and which was further increased after inhibiting the expression of miR-124-3p. Furthermore, the inhibition of miR-124-3p decreased the protein expression level of E-ca and increased the expression of VIM, α-SMA, N-ca, FN, COL1, MMP2 and MMP9, thus exacerbating the process of EMT and fibrosis in HK2/NRK-49F cells treated by TGF-β1 (Fig. 4j–l). Taken together, these data indicated that miR-124-3p is regulated the process of EMT and fibrosis via targeting ITGB1 in TGF-β1 treated HK2 and NRK-49F cells.

### lncRNA MALAT1 regulates the expression level of ITGB1 in TGF-β1 induced HK2/NRK-49F cells fibrosis process by inhibiting miR‐124‐3p

**Figure 5:**
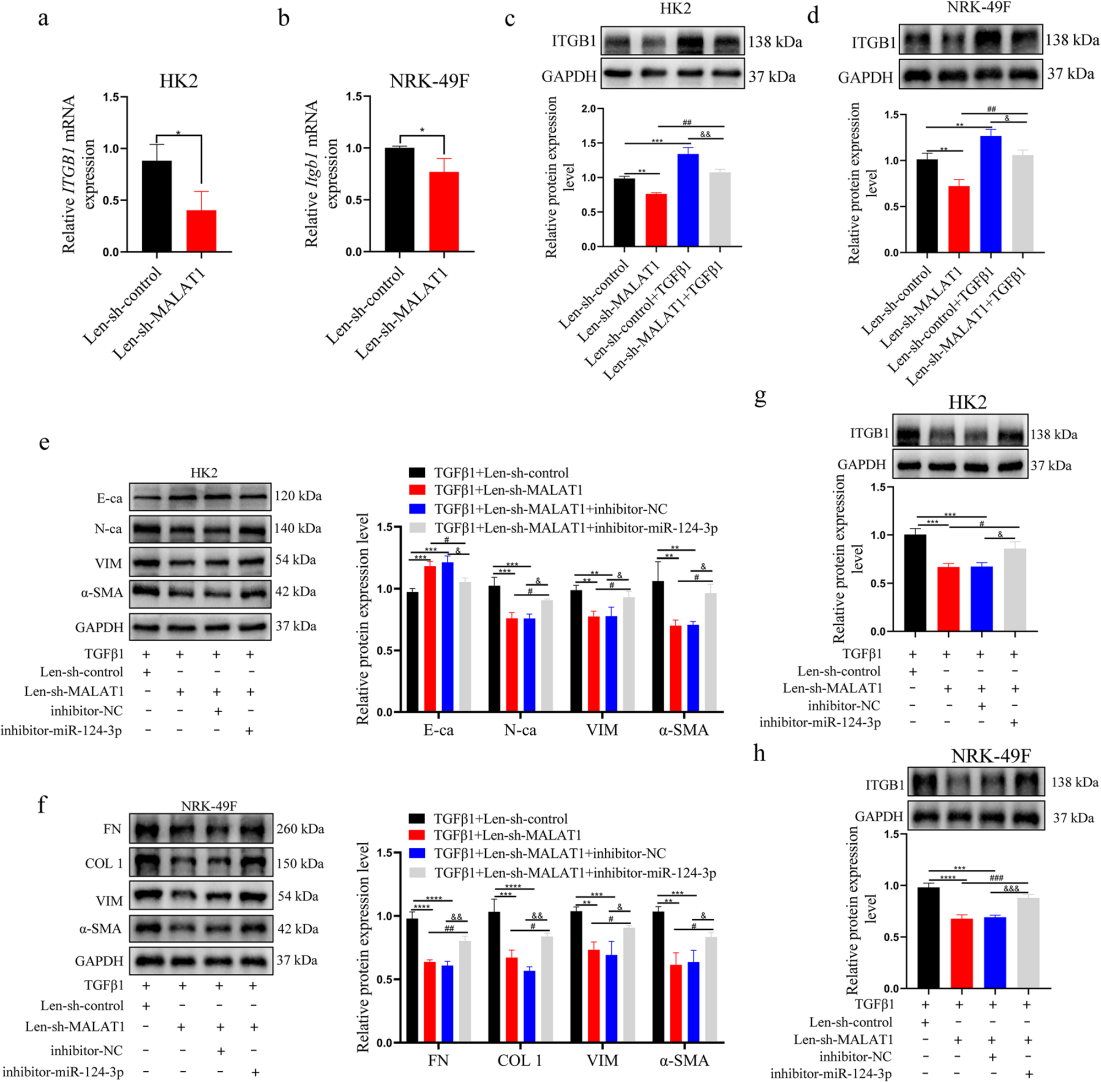
(**a**, **b**) qRT-PCR was used to detect the mRNA expression of ITGB1 after transfection with Len-sh-MALAT1 in HK2 and NRK-49F cells. *: *p* < 0.05; (**c**, **d**) After Len-sh-MALAT1 was transfected into HK2 and NRK-49F cells treated with or without TGF-β1, the expression level of ITGB1 protein was detected by WB (from 3 experiments). **: *p* < 0.01; ***: *p* < 0.001 (Len-sh-MALAT1group and Len-sh-control + TGF β1 group compare with Len-sh-control); ##: *p* < 0.01 (Len-sh-MALAT1 group was compared with Len-sh-MALAT1 + TGF β1group); &: *p* < 0.05; &&: *p* < 0.01 (Len-sh-control + TGF β1 group was compared with Len-sh-MALAT1 + TGF β1 group) (**e**, **f**, **g**, **h**) After co-transfected with inhibitor-miR-124-3p and Len-sh-MALAT1 in TGF-β1 induced HK2 and NRK-49F cells, ITGB1 and fibrosis-related protein expression were assessed by WB (from 3 experiments). **: *p* < 0.01; ***: *p* < 0.001; ****: *p* < 0.0001 (TGF β1 + Len-sh-MALAT1 group and TGF β1 + Len-sh-MALAT1 + inhibitor-NC were compare with TGF β1 group); #: *p* < 0.05; ##: *p* < 0.01; ###: *p* < 0.001 (TGF β1 + Len-sh-MALAT1 + inhibitor-miR-124-3p group was compare with TGF β1 + Len-sh-MALAT1group); &: *p* < 0.05; &&: *p* < 0.01; &&&: *p* < 0.001 (TGF β1 + Len-sh-MALAT1 + inhibitor-miR-124-3p group was compare with TGF β1 + Len-sh-MALAT1 + inhibitor-NC group).

To further verify the lncRNA MALAT1/miR-124-3p/ITGB1 regulatory network in RIF, the alteration of lncRNA MALAT1 and miR‐124‐3p expression was performed simultaneously. Firstly, we demonstrated that lncRNA MALAT1 knockdown attenuated the mRNA and protein level of ITGB1 in the two cell lines (Fig. 5a–d). Subsequently, HK2 and NRK-49F cells were simultaneously co-transfected with Len-sh-MALAT1 and inhibitor-miR-124-3p and then treated by TGF-β1. After that, the expression levels of renal fibrosis-related markers were determined by WB assays. Results showed that silencing lncRNA MALAT1 expression significantly increased the expression of E-ca and decreased the expression level of fibrosis-related markers. Moreover, the protect effect of Len-sh-MALAT1 on EMT and fibrosis protein levels could be partially reversed by co-transfected with inhibitor-miR-124-3p (Fig. 5e, f). Furthermore, the protein expression trend of ITGB1 was consistent with that of fibrosis-related markers (Fig. 5g, h). These results revealed that lncRNA MALAT1 regulates the level of ITGB1 and TGF-β1 induced HK2/NRK-49F cells fibrosis process could be partially reversed by inhibitor-miR-124-3p.

### lncRNA Malat1 knockdown alleviate the degree of fibrosis in UUO mice model

**Figure 6:**
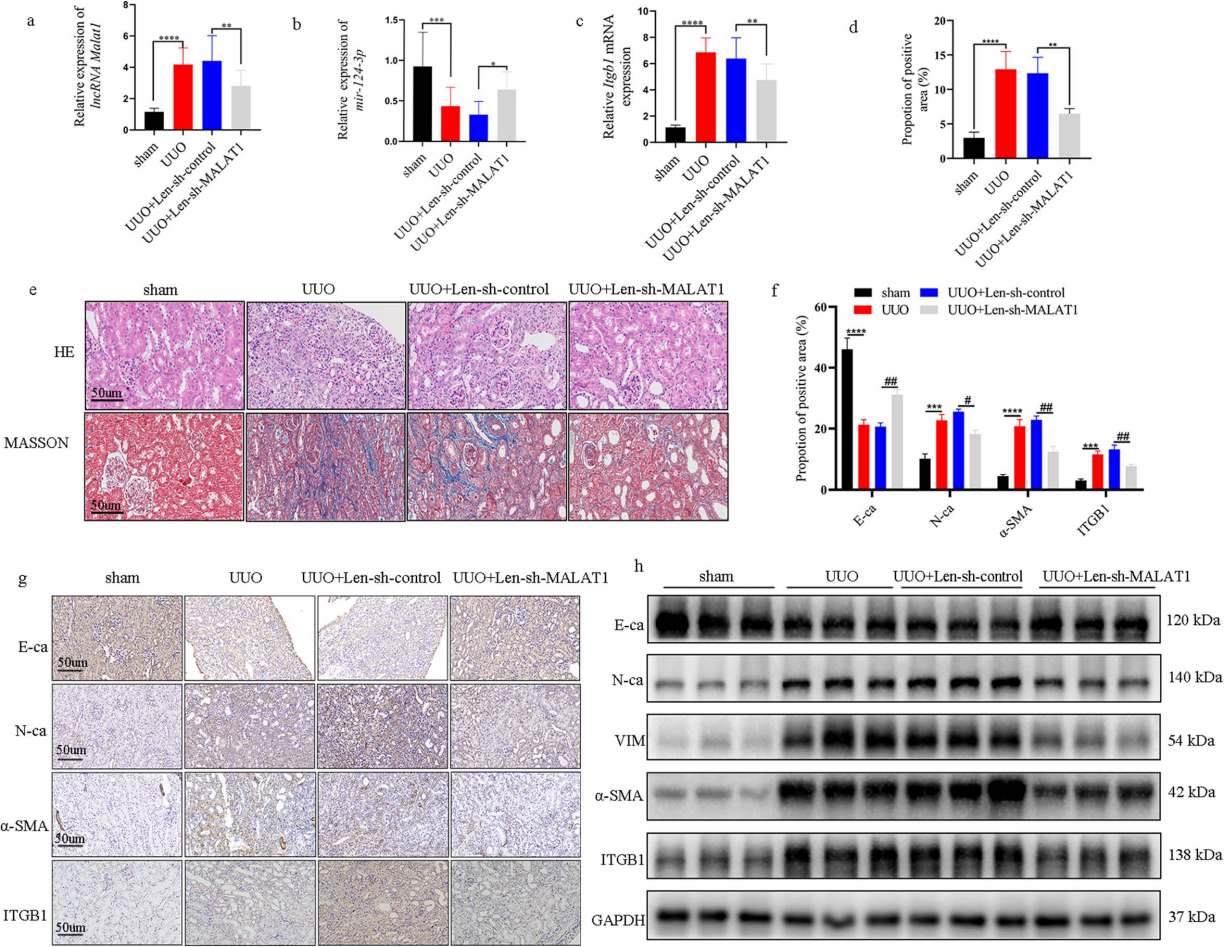
(**a**, **b**, **c**) The expression level of lncRNA Malat1, mir-124-3p, Itgb1 were assessed by qRT-PCR in UUO and UUO + Len-sh-MALAT1 mice model. *p < 0.05; **: *p* < 0.01; ***: *p* < 0.001; ****: *p* < 0.0001. (**d**, **e**) Samples of renal tissue stained with HE, and Masson’s trichrome (bar = 50um). ImageJ image analysis revealing that the percentage of the positive area of collagen in each group (n = 4). **: *p* < 0.01; ****: *p* < 0.0001. (**f**, **g**) IHC staining for ITGB1, E-ca, N-ca, α-SMA expression in UUO and UUO + Len-sh-MALAT1 mice model (bar = 50 um). semi-quantitative data showing the relative expression of E-ca, N-ca, α-SMA and ITGB1 in each group (n = 4). ***: *p* < 0.001; ****: *p* < 0.0001 (compare with sham group). #: *p* < 0.05; ##: *p* < 0.01 (compare with UUO + Len-sh-control group). (**h**) WB was performed to determine the protein expression of E-ca, N-ca, VIM, α-SMA, ITGB1 in renal tissues (n = 3).

To further verify our in vitro findings, we established UUO mice model to confirm the lncRNA Malat1 effect in vivo. The relative mRNA expression trend of lncRNA Malat1, mir-124-3p and Itgb1 in the kidney of UUO mice were consistent with the results of cellular level (Fig. 6a–c). The HE staining results were observed that kidney tissues underwent a series of typical pathological changes after ligation of the ureter. In contrast, transfection with Len-sh-MALAT1 led to less pathological damage compared to transfection with Len-sh-control in the UUO mice kidney (Fig. 6e). Moreover, Masson trichrome staining revealed that there were more deposits of collagen (blue color) in the UUO model than in the sham operation group. However, less deposits of collagen were observed in the UUO model transfected with Len-sh-MALAT1 compared with UUO + Len-sh-control group (Fig. 6d, e). As shown in Fig. 6f, g, the IHC results demonstrated that the expression of epithelial marker E-ca was suppressed and the mesenchymal markers (α-SMA, N-ca), and ITGB1 were induced in UUO mice model. However, when the UUO mice were injected with Len-sh-MALAT1, fibrosis process and ITGB1 were significantly repressed. WB analysis results for E-ca, α-SMA, and N-ca, VIM, ITGB1 were also in consistency with the IHC data (Fig. 6h). In summary, these results revealed that lncRNA Malat1 knockdown alleviated RIF is associated with the suppression of EMT and fibrosis process in vivo based on evaluation of several proteins.

### Expression of lncRNA MALAT1/miR-124-3p/ITGB1 axis and fibrosis-related markers in renal tissue samples of ON patients

**Figure 7:**
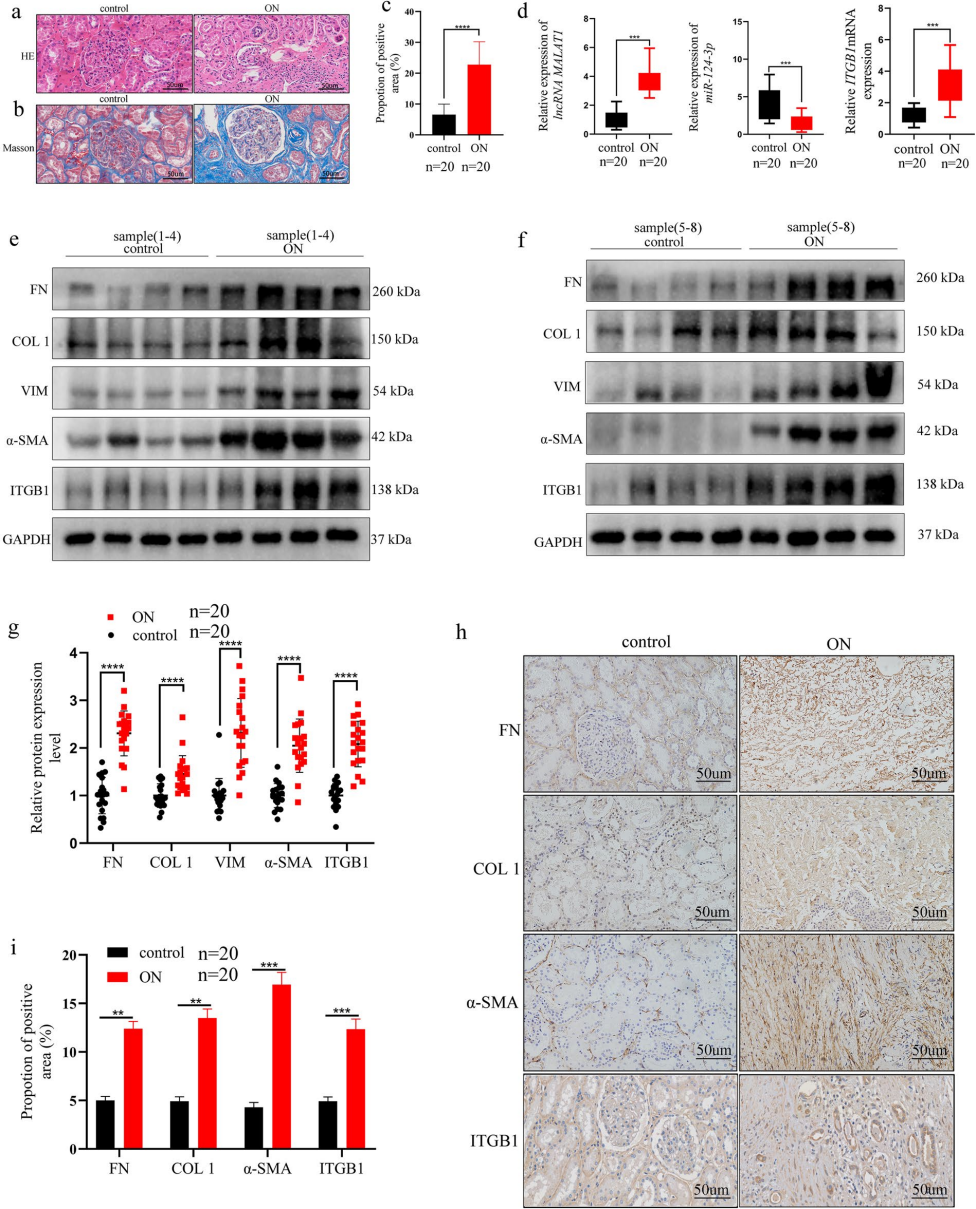
(**a**) Pathological changes were analyzed using HE staining in ON induced RIF clinic samples(bar = 50 um). (**b**, **c**) The extent of collagen deposition in RIF was assessed with Masson staining (bar = 50 um). ImageJ image analysis revealing that the percentage of the positive area of collagen deposition in clinical kidney specimen (n = 20). ****: *p* < 0.0001 (**d**) qRT-PCR showed that lncRNA MALAT1and ITGB1 were upregulated in ON induced RIF clinic samples, while miR-124-3p was downregulated. ***: *p* < 0.001. (**e**, **f**, **g**) WB was performed to determine the protein expression of FN, COL 1, VIM, α-SMA, ITGB1 in clinic renal tissues. ****: *p* < 0.0001 h, (**i**) IHC staining for FN, COL 1, α-SMA, ITGB1expression in clinic renal tissues(bar = 50 um). semi-quantitative data showing the relative expression of FN, COL 1, α-SMA and ITGB1 in each group (n = 20). **: *p* < 0.01; ***: *p* < 0.001.

To assess the expression of lncRNA MALAT1/miR-124-3p/ITGB1 axis and fibrosis-related markers in ON patients, HE staining, Masson staining, qRT-PCR for RNA analysis, WB, and IHC were performed. HE staining revealed that renal damage was more serious compared to the control group (Fig. 7a). Moreover, Masson trichrome staining showed extensive collagen deposition in the renal interstitial of ON group (Fig. 7b). The positive area of the two groups was quantified and the difference was statistically significant (Fig. 7c). Furthermore, the qRT-PCR results showed that the mRNA expression level of lncRNA MALAT1 and ITGB1 were significantly increased, while miR-124-3p expression level were downregulated in ON compared to control group (Fig. 7d). Notably, WB results showed that FN, α-SMA, VIM, COL1 and ITGB1 were upregulated in ON group compared with control group (Fig. 7e–g). Additionally, IHC also confirmed that the positive expression of fibrosis-related markers (FN, COL1, α-SMA) and ITGB1 were significantly increased in the ON group (Fig. 7h, i). Thus, we concluded that lncRNA MALAT1/miR-124-3p/ITGB1 axis may closely related to the ON induced RIF.

## Discussion

To date, RIF remains a major public health problem. The exploration of early specific diagnostic markers and appropriate treatment play an important role in preventing the development of ESRD from renal fibrosis. Multiple types of cells, including fibroblasts, epithelial cells, endothelial cells and pericytes, contribute to RIF formation, and lots of mediators participate in the pathological process of fibrosis under different disease conditions. For example, TGF-β1 has long been recognized as a key mediator of RIF and induces renal pathological changes largely by activating its downstream Smad signaling pathway^41^. High glucose induced the production of multiple proinflammatory cytokines (e.g., IL-1, IL-6, and TNF-α), ECM proteins^42^, autophagic flux in HK2 cells^43^. High glucose often plays a key role in the development of diabetic nephropathy, and a growing body of evidence suggests that dysregulation of NLRP3 activation may play a role in the progress of diabetic nephropathy via regulation of inflammatory response and RIF^44,45^. As a renal growth factor, angiotensin (Ang) II could also increases proinflammatory process and regulates matrix degradation in RIF^46,47^. Thus, regulation of these mediators are probably one of the promising targets for treating RIF.

As functional RNA molecules, lncRNAs act as crucial roles in regulating specific cellular processes, such as modulate protein-coding gene expression in the transcriptional, and post-transcriptional, epigenetic levels^17,18^. Recently, with the development of high-throughput sequencing technology, a growing number of studies have discovered that many lncRNAs and miRNAs involved in the development of RIF by ceRNA mechanism, thereby affecting gene expression by regulating mRNA stability, degradation, and translation^48–50^. For instance, down-regulation of lncRNA GAS5 had an antifibrosis effect in vitro and in vivo by regulation of miR-96-5p/FN1 axis^50^. Furthermore, lncRNA NEAT1 sponged miR-129 to modulate the EMT process and inflammation response of renal fibrosis by regulating collagen I expression^51^. lncRNA MALAT1, one of the first discovered lncRNAs, has been widely studied in breast cancer metastasis, lung cancer, colorectal cancer and other tumor diseases^52–54^. However, the understanding of lncRNA MALAT1 function and mechanisms in RIF is remains limited. In this study, we uncovered a pathogenic role of lncRNA MALAT1 in UUO mice model and also elucidated its potential molecular mechanism by using HK2 and NRK-49F cells treated by TGF-β1.

Studies found that EMT is closely related to kidney tubular epithelial cells and occurs in early stage of renal fibrosis^55^. Moreover, excessive deposition of ECM in the tubulointerstitial is also the most important characteristic in RIF, which ultimately leads to renal dysfunction^56^. Combined with the studies on lncRNA MALAT1 in other fibrosis diseases^22,57–59^, we suggest that lncRNA MALAT1 is one member of pro-fibrosis lncRNAs, which may also play an important role in the development of RIF. To further investigate the function of lncRNA MALAT1 in RIF disease. Initially, we demonstrated that the expression of lncRNA MALAT1 was significantly increased both in HK2 and NRK-49F cells treated by TGF-β1 and in UUO mice model, which are consistent with the previous reported results by Liu et al.^27^. Furthermore, we also found that lncRNA MALAT1 was significantly increased in clinical sample in our study. In addition, we deleted lncRNA MALAT1 expression by transfected Len-sh-MALAT1 in vitro and in vivo. As a result, EMT process and mesenchymal markers were obviously inhibited. The above results indicate that lncRNA MALAT1 also acts as a pro-EMT and pro-fibrosis in RIF. Simultaneously, we identified that the subcellular localization of lncRNA MALAT1 was localized both in the cytoplasm and in the nucleus in HK2 and NRK-49F cells by using FISH method, mainly in cytoplasm, indicating that its potential ceRNA mechanism in gene regulation.

Previous study indicated that miRNAs have emerged as pivotal gene regulators in diverse biological pathways^60^. Recently, miR-124-3p is one of common studied miRNA in fibrosis diseases. For example, research demonstrated that rosiglitazone inhibited the activation of hepatic stellate cells by upregulating miR-124-3p expression, thereby alleviating liver fibrosis^61^. By analyzing RNA-sequencing data, researcher also found that miR-124-3p played an important role in cardiac fibrosis process, which suggest effective future therapeutics for cardiac fibrosis^28^. We also found that miR-124-3p played an important role in TGF-β1 induced EMT activation of HK2 cells, but did not affect cell proliferation process. At the same time, inhibition of miR-124-3p expression in TGF-β1 induced NRK-49F cells promoted cell proliferation, migration and cell transformation process. Therefore, miR-124-3p may participate in various stages of renal interstitial fibrosis process. Moreover, ceRNA can serve as an important regulatory mechanism in a variety of fibrosis diseases. Using starBase v3.0 software predictive analysis, we aslo predicted that miR-124-3p is a putative target of lncRNA MALAT1. In our study, our results already indicated that lncRNA MALAT1 directly bound to miR-124-3p. More intriguingly, qRT-PCR outcomes also demonstrated that the expression of miR-124-3p was suppressed in HK2 and NRK-49F cells treated by TGF-β1, which was enhanced by lncRNA MALAT1 knockdown.

lncRNAs, serve as ceRNA, regulate miRNAs expression and then affect the depression of corresponding target gene through post-transcriptional regulation^20^. Previous studies reported that miR-124-3p directly binds to 3′UTR of ITGB1 mRNA, and ITGB1 expression was inversely related to miR-124-3p in NRK-52E cells^62^. Meanwhile, our results showed that the expression of ITGB1 was dramatically attenuated by mimics-miR-124-3p. Furthermore, inhibition expression of miR-124-3p could significantly upregulated the mRNA and protein level of ITGB1 in HK2 and NRK-49F cells. In addition, lncRNA MALAT1 silencing reduces the protein levels of ITGB1, fibrosis-related markers in UUO mice model and in HK2/NRK-49F cells treated by TGF-β1, and inhibition of miR-124-3p obviously reversed the effects of lncRNA MALAT1 downregulation exerted in vitro. Meanwhile, the expression of lncRNA MALAT1/miR-124-3p/ITGB1 and the fibrosis-related markers in ON renal specimens were consistent with the expression trend in cell and animal experiments. Taken together, we indicate that lncRNA MALAT1 might play its function by regulating ITGB1 via miR-124-3p sponging in RIF process.

In conclusion, our results show that TGF-β1 induce the expression of lncRNA MALAT1. Interestingly, knockdown of lncRNA MALAT1 not only alleviates the fibrosis process in the two cell lines treated by TGF-β1, but also has been verified in the UUO mice model. Mechanistically, lncRNA MALAT1 directly interacts with miR-124-3p to regulate ITGB1 expression, thus leading to the promotion of RIF in vitro and in vivo. To sum up, our study suggested that silencing of lncRNA MALAT1 plays an anti-fibrotic role, which may serve as an effective future therapeutic target for RIF.

### Supplementary Information

Supplementary Figures.

Supplementary Figure 1.

Supplementary Figure 2.

Supplementary Legends.

Supplementary Table 1.

## Data Availability

The datasets generated and/or analysed during the current study are available from the corresponding author on reasonable request.
