# Supplementary material for: Knockdown of lncRNA MALAT1 attenuates renal interstitial fibrosis through miR-124-3p/ITGB1 axis
Source: Sci Rep. 2023 Oct 23;13:18076. doi: 10.1038/s41598-023-45188-y (PMC10593763; doi:10.1038/s41598-023-45188-y)
Supplement: Supplementary file 1 — Supplementary Figures. [file 41598_2023_45188_MOESM1_ESM.pdf]

Supplementary Fig.2a

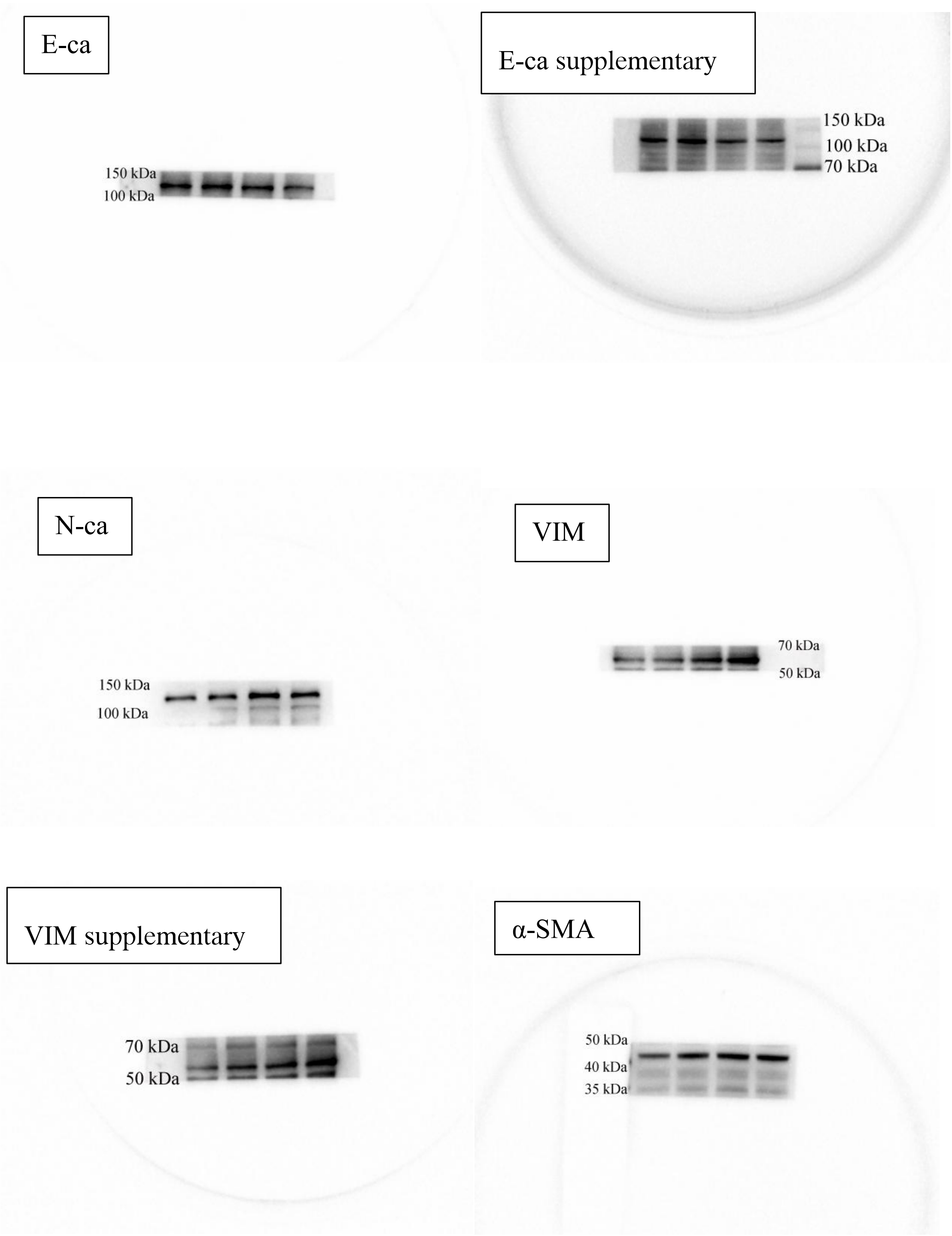

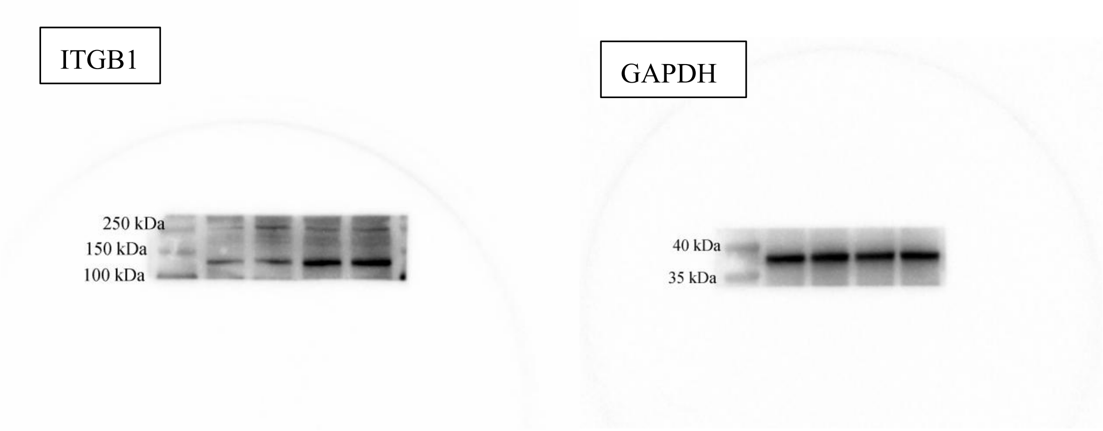

Supplementary Fig.2b

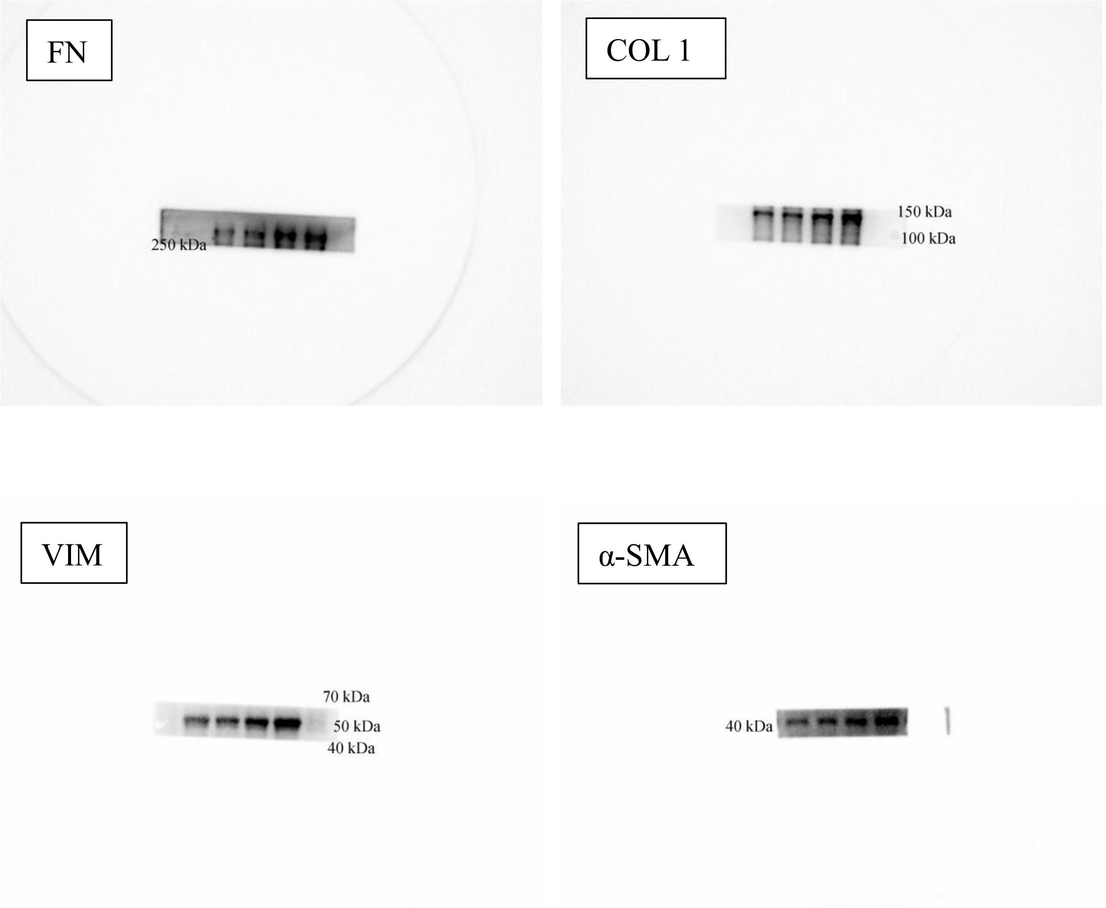

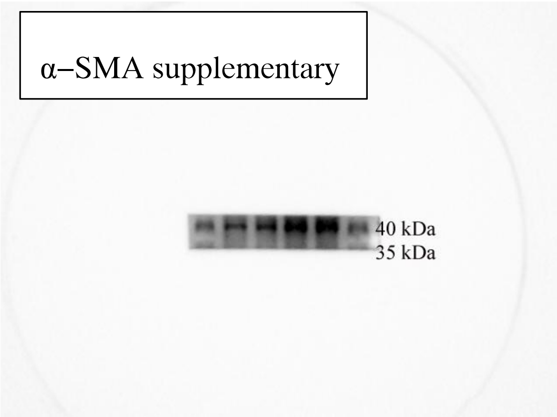
α−SMA supplementary
40 kDa
35 kDa

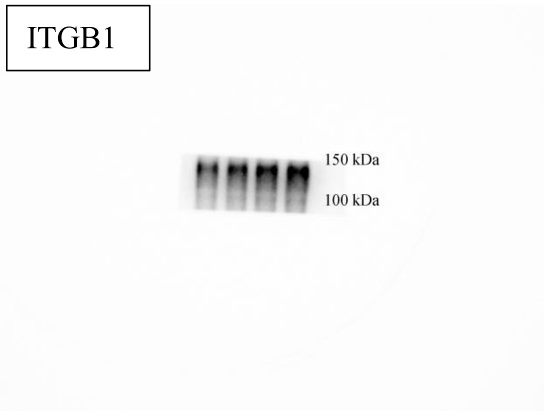
ITGB1
150 kDa
100 kDa

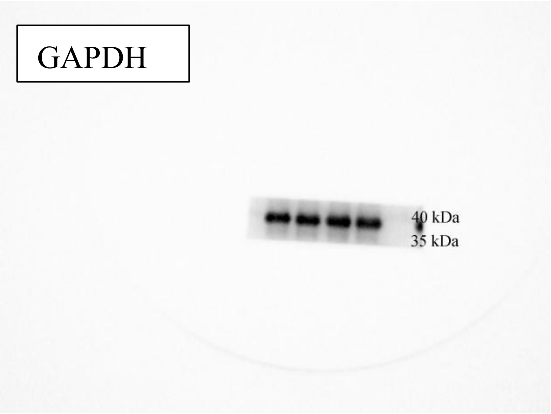
GAPDH
40 kDa
35 kDa

Fig.2c

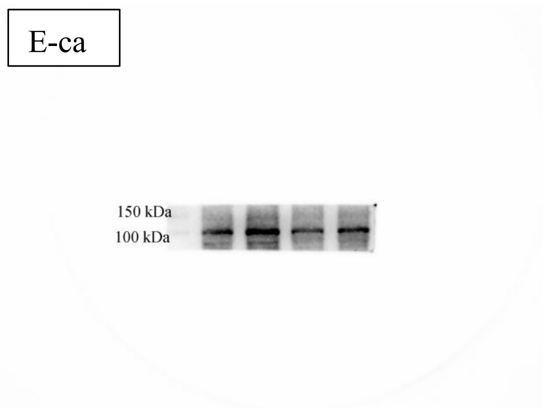
E-ca
150 kDa
100 kDa

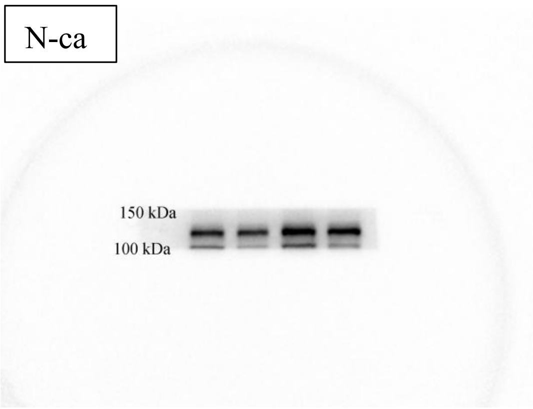
N-ca
150 kDa
100 kDa

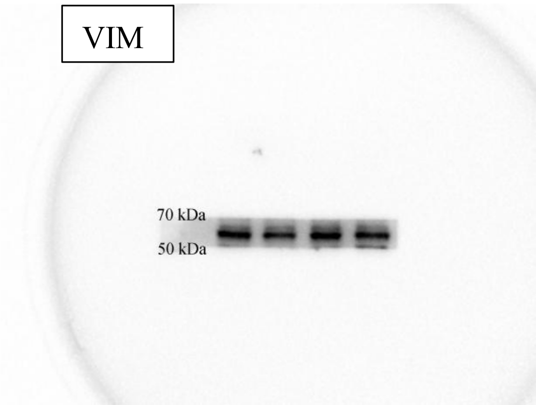
VIM
70 kDa
50 kDa

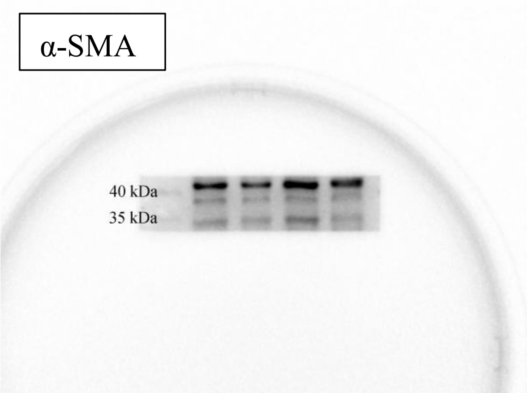
α-SMA
40 kDa
35 kDa

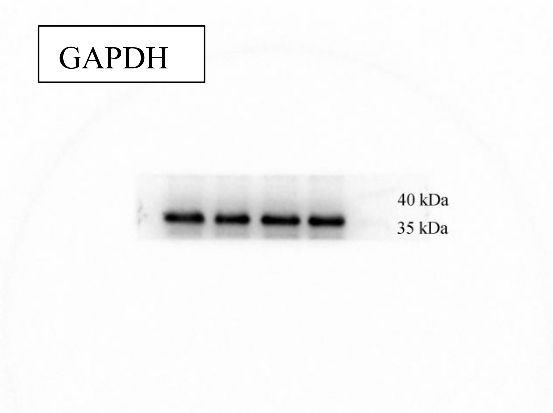
GAPDH
40 kDa
35 kDa

Fig.2d

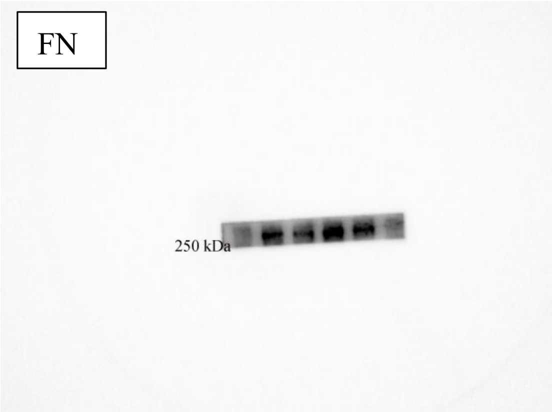
FN
250 kDa

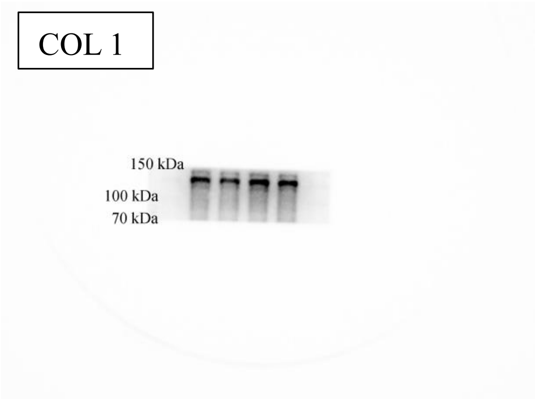
COL 1
150 kDa
100 kDa
70 kDa

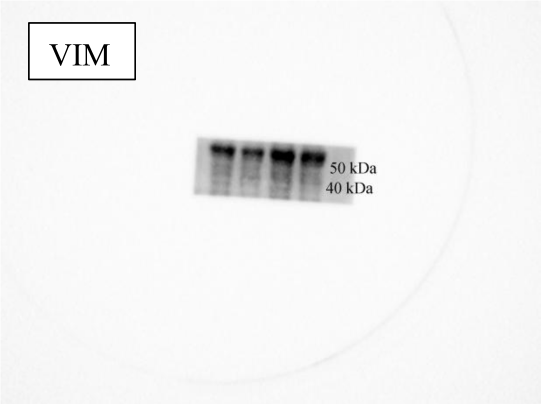
VIM
50 kDa
40 kDa

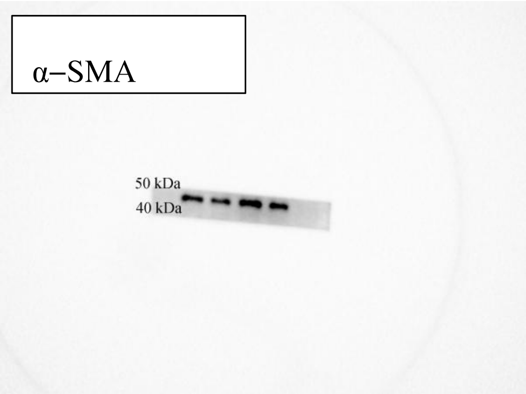
α−SMA
50 kDa
40 kDa

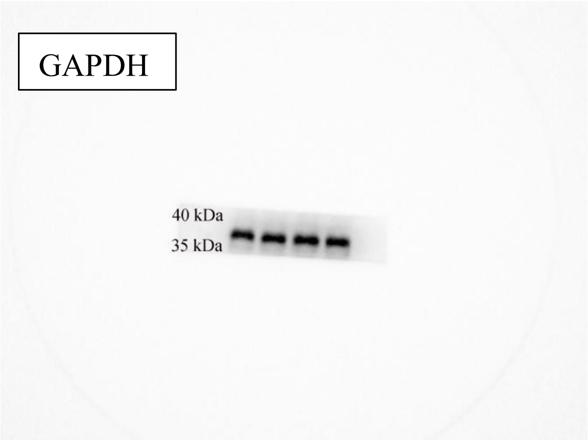
GAPDH
40 kDa
35 kDa

Fig.3e

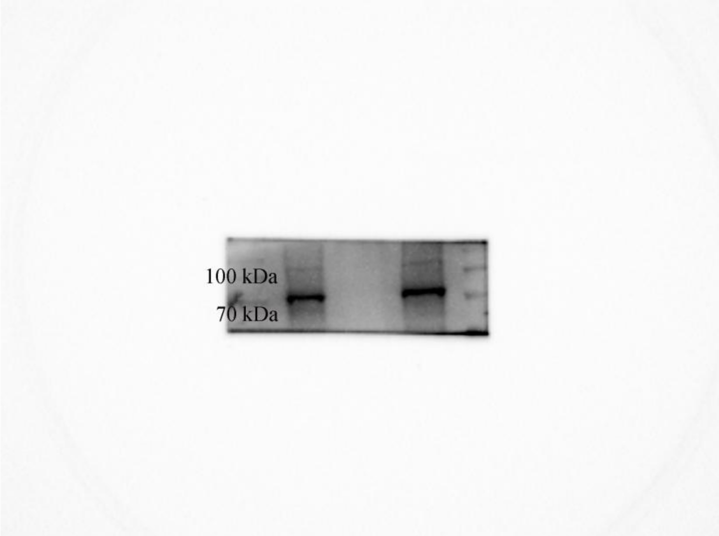
100 kDa
70 kDa

Fig.3f

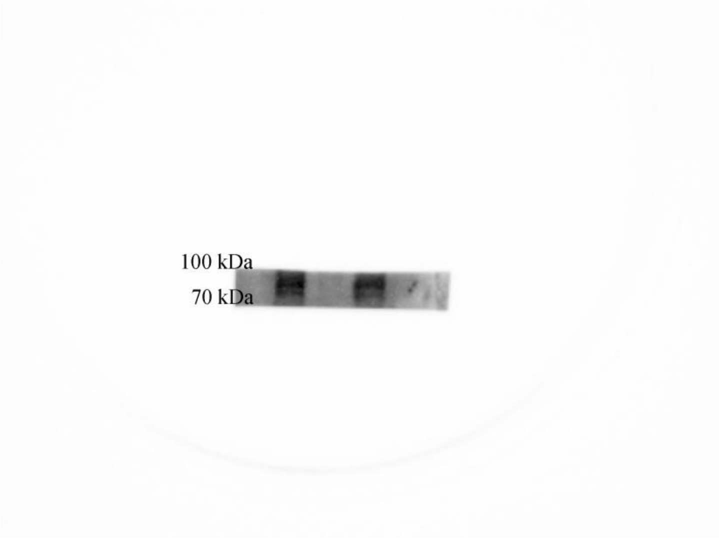
100 kDa
70 kDa

Fig.4j

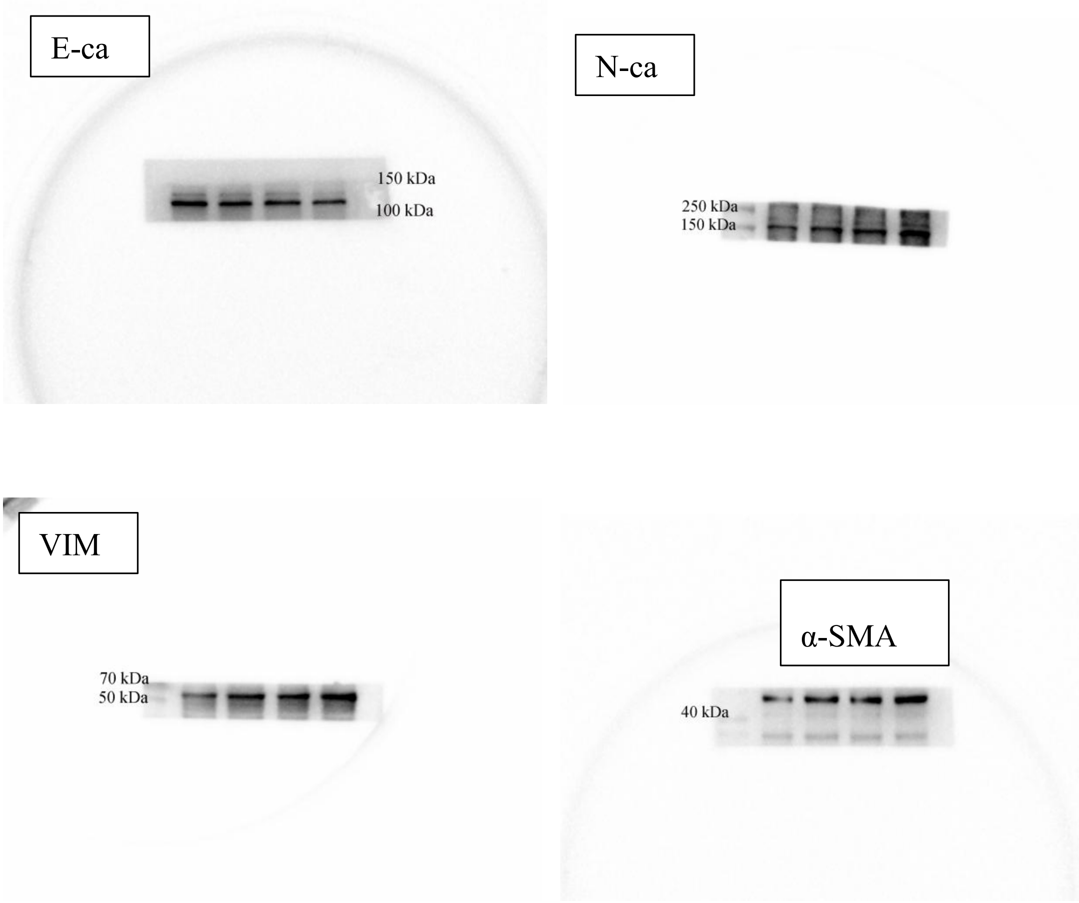
E-ca
150 kDa
100 kDa
N-ca
250 kDa
150 kDa
VIM
70 kDa
50 kDa
α-SMA
40 kDa

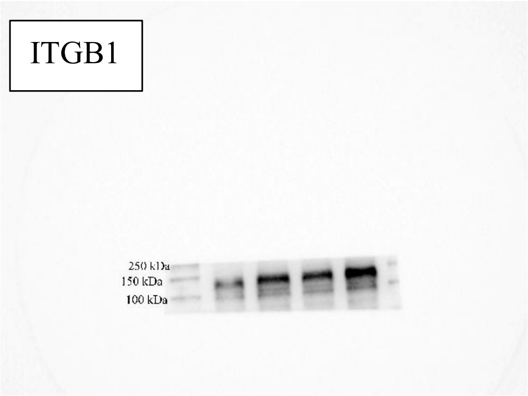
ITGB1
250 kDa
150 kDa
100 kDa

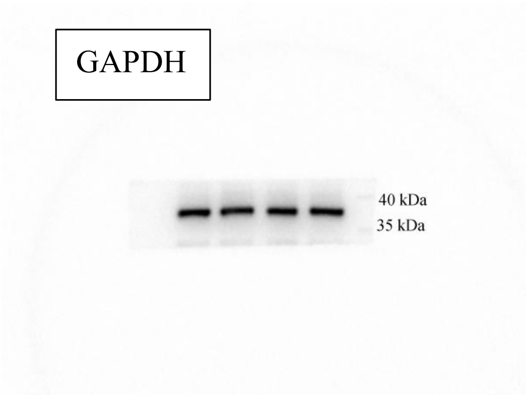
GAPDH
40 kDa
35 kDa

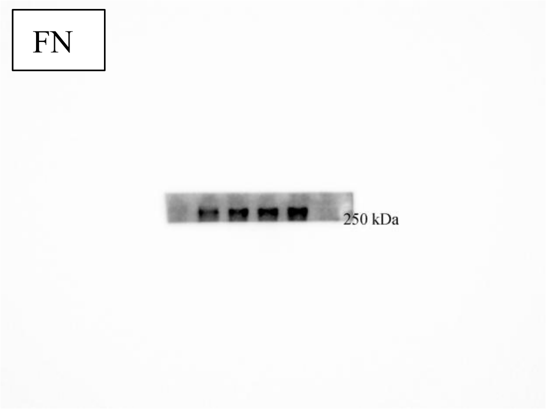
FN
250 kDa

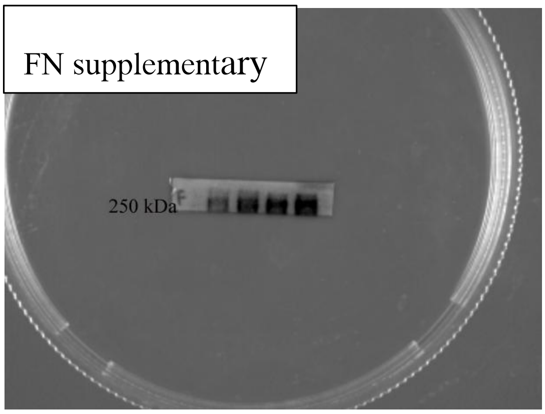
FN supplementary
250 kDa

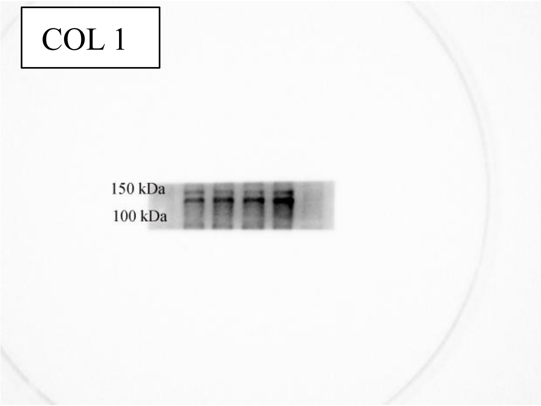
COL 1
150 kDa
100 kDa

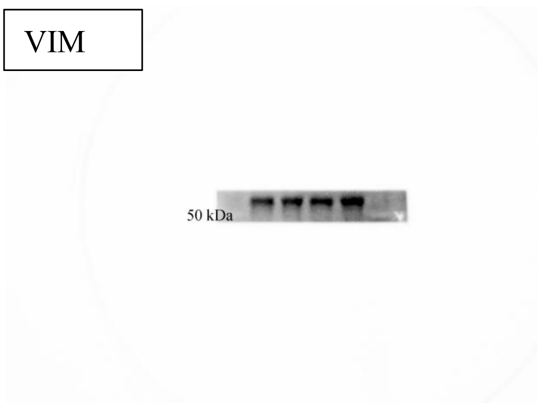
VIM
50 kDa

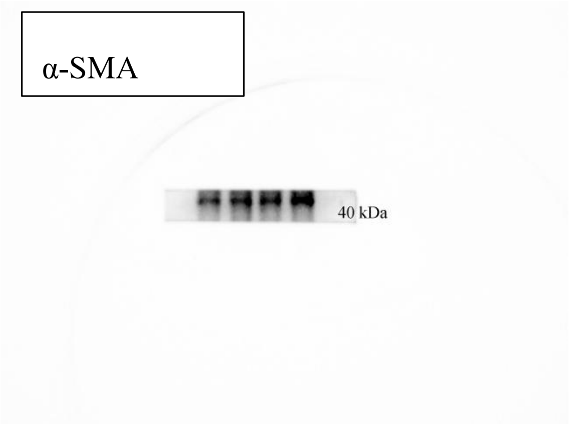
α-SMA
40 kDa

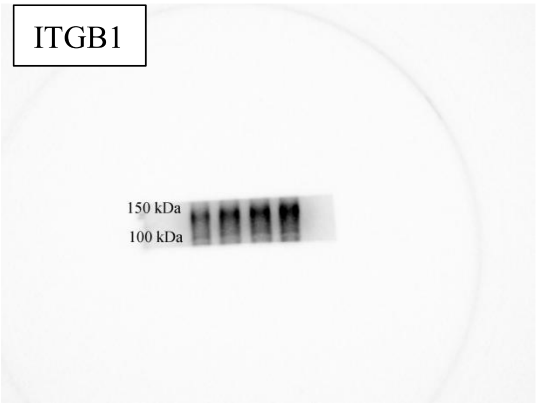
ITGB1
150 kDa
100 kDa

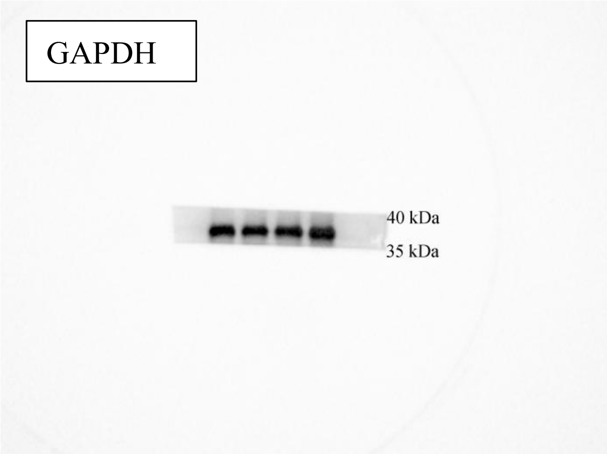
GAPDH
40 kDa
35 kDa

Fig.4k

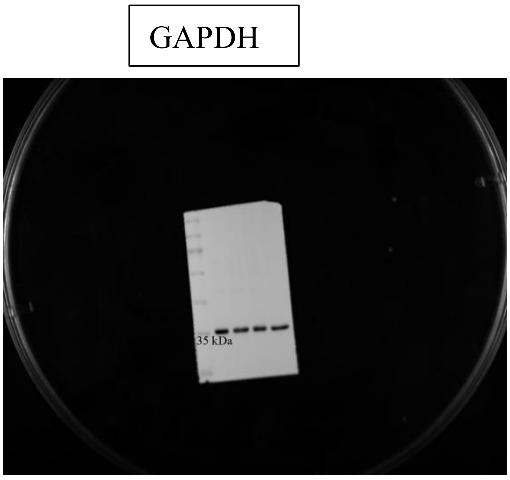
GAPDH
35 kDa

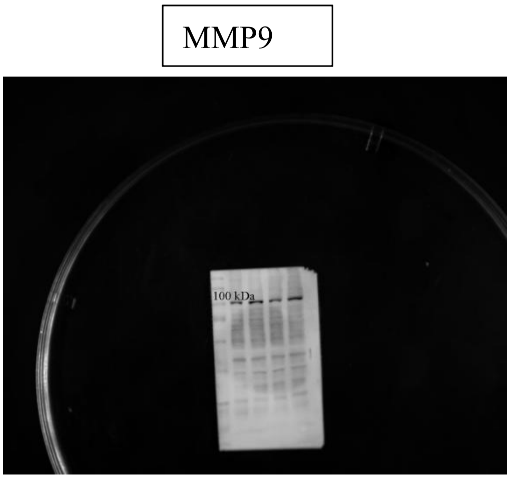
MMP9
100 kDa

MMP2

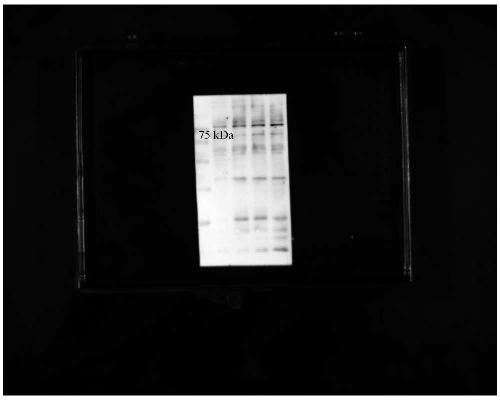

Fig.4l

GAPDH

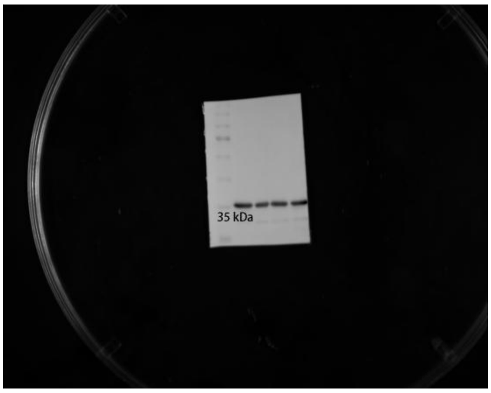

MMP9

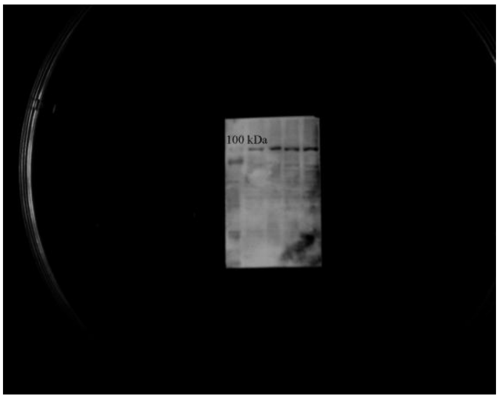

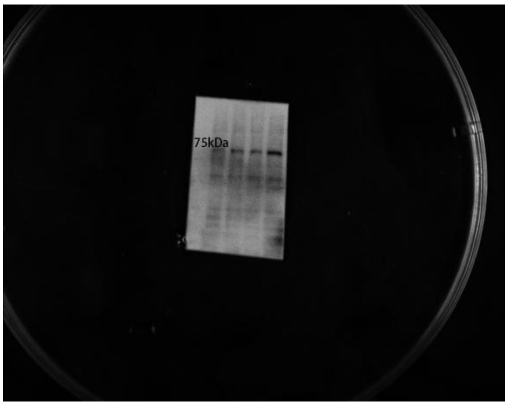

MMP2

Fig.5c

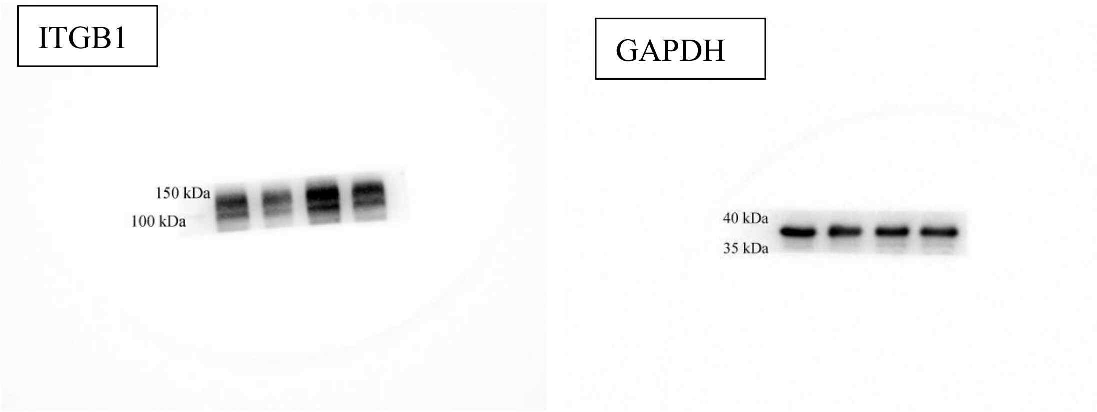
ITGB1
150 kDa
100 kDa
GAPDH
40 kDa
35 kDa

Fig.5d

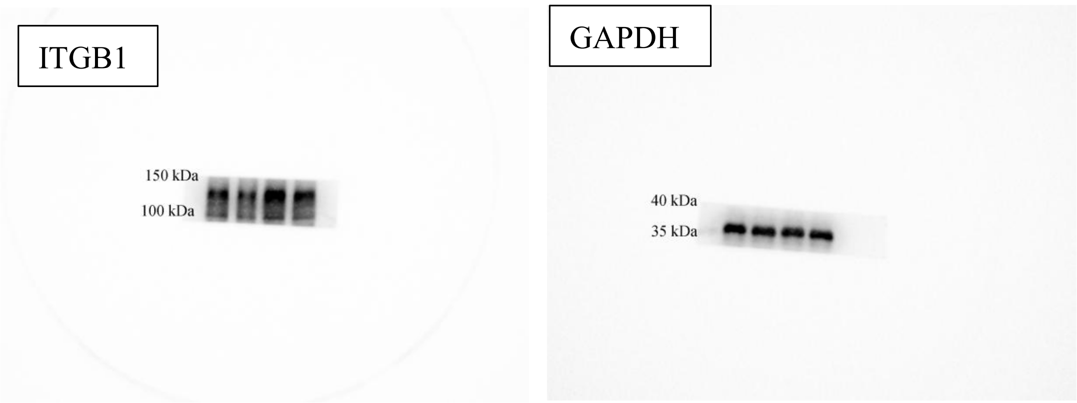
ITGB1
150 kDa
100 kDa
GAPDH
40 kDa
35 kDa

Fig.5e

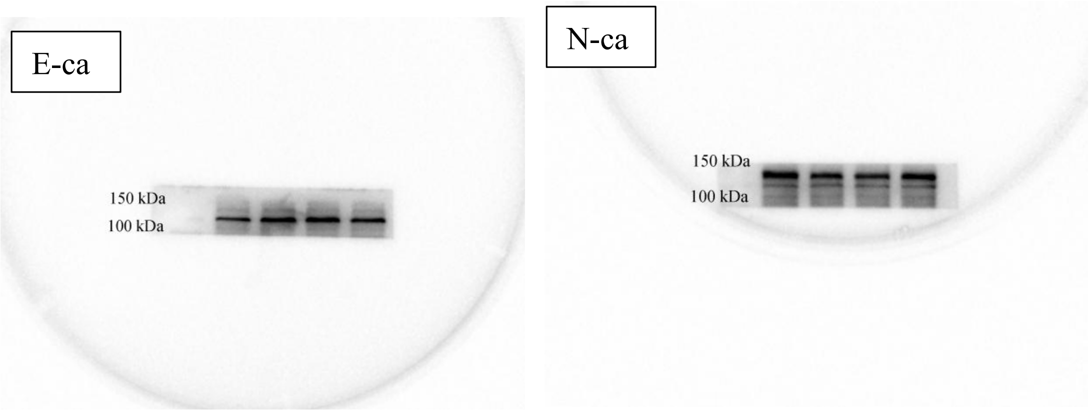
E-ca
150 kDa
100 kDa
N-ca
150 kDa
100 kDa

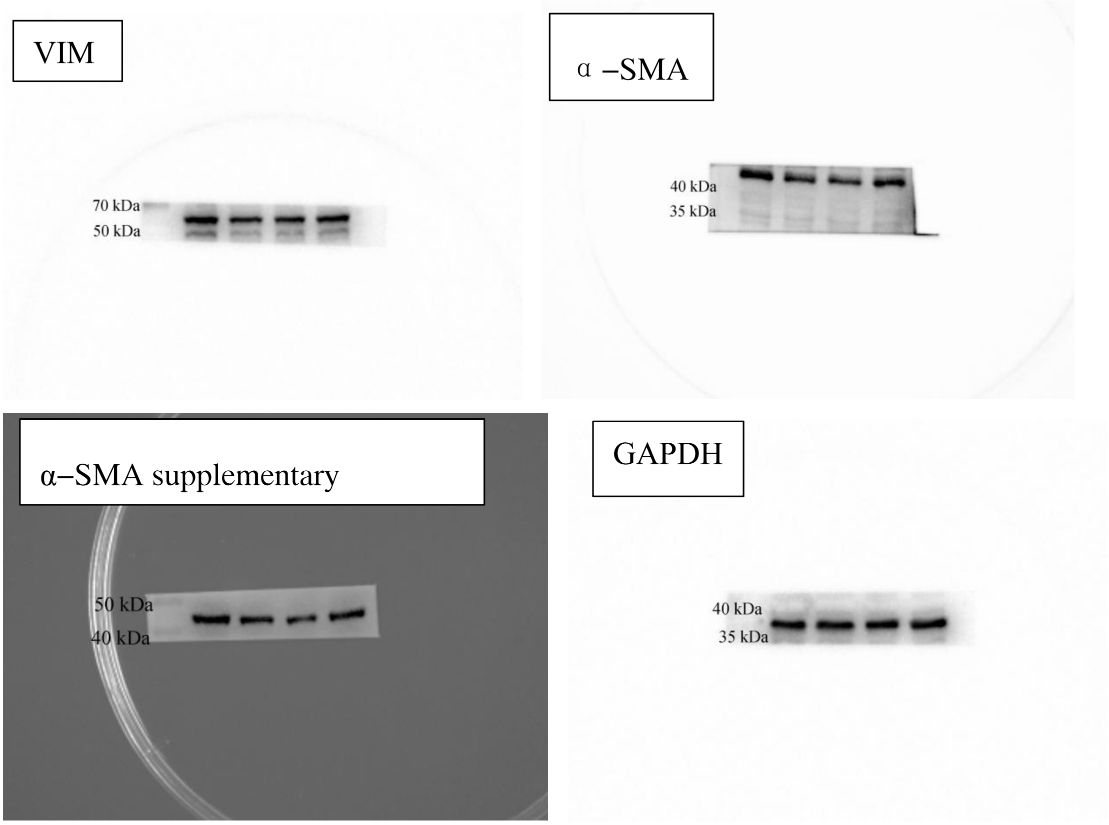
VIM
70 kDa
50 kDa
α –SMA
40 kDa
35 kDa
α–SMA supplementary
50 kDa
40 kDa
GAPDH
40 kDa
35 kDa

Fig.5f

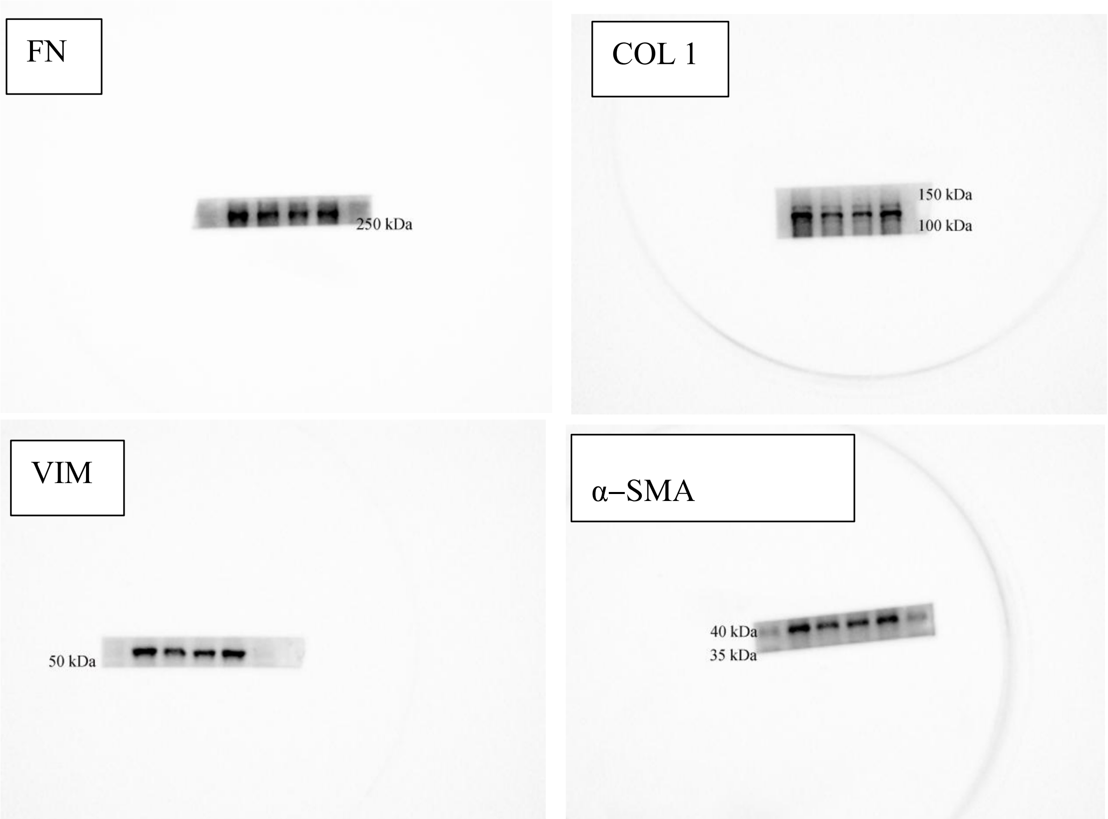
FN
250 kDa
COL 1
150 kDa
100 kDa
VIM
50 kDa
α–SMA
40 kDa
35 kDa

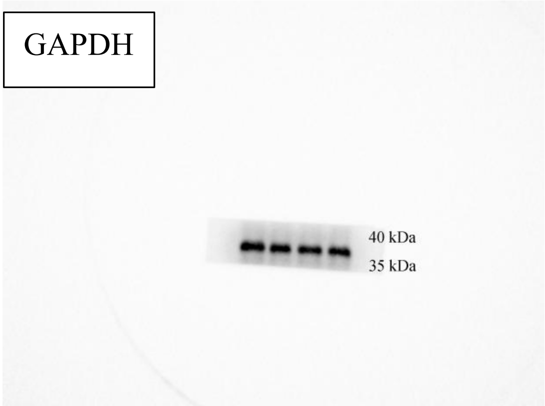

Fig.5g

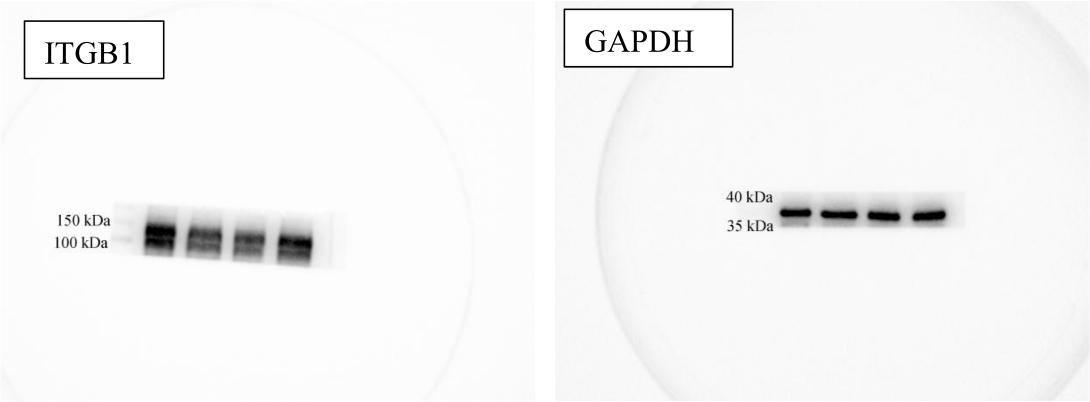

Fig.5h

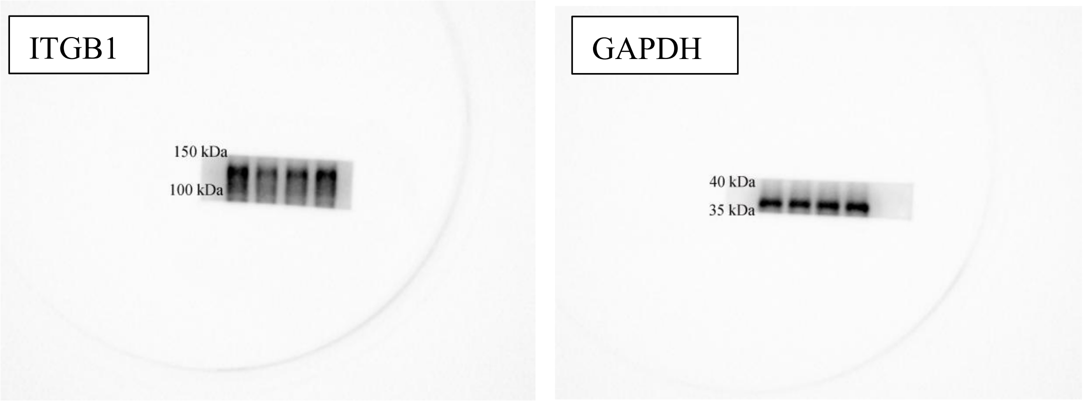

Fig.6h

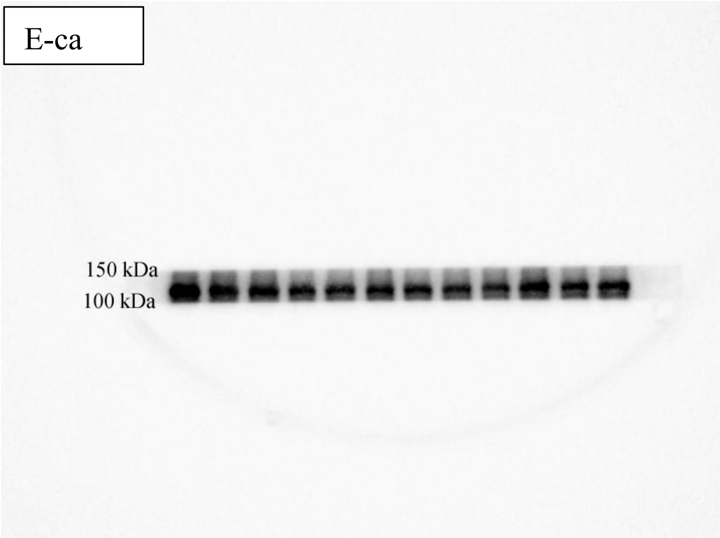
E-ca
150 kDa
100 kDa

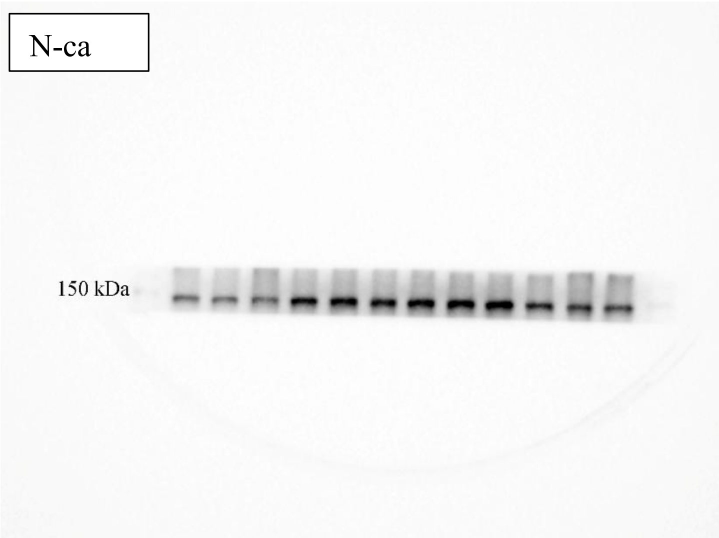
N-ca
150 kDa

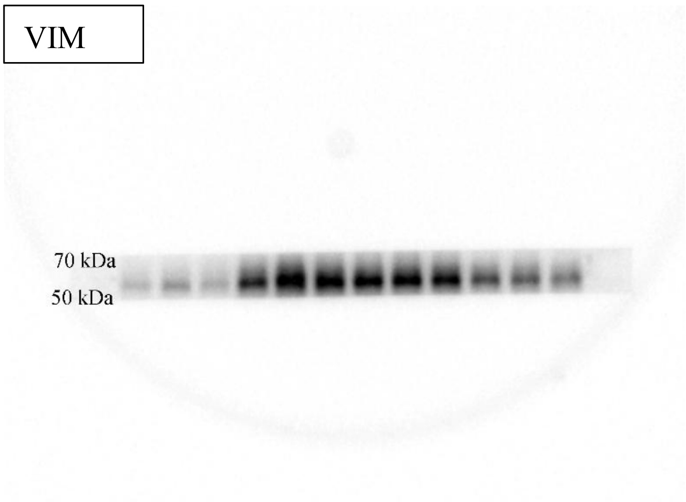
VIM
70 kDa
50 kDa

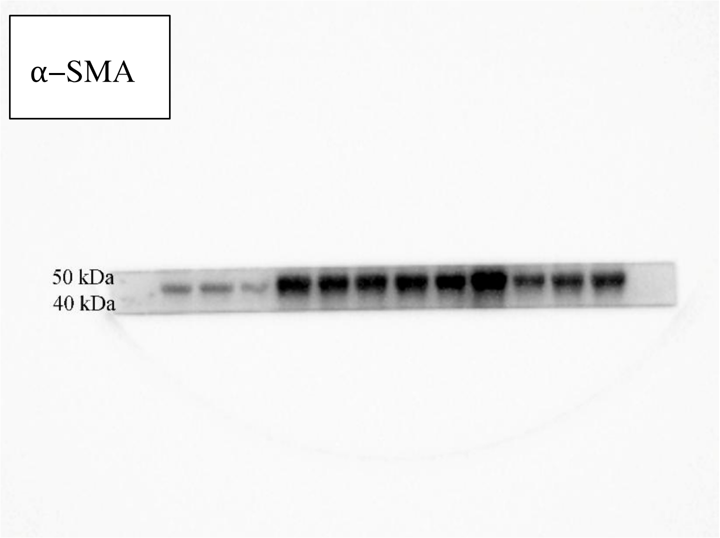
α–SMA
50 kDa
40 kDa

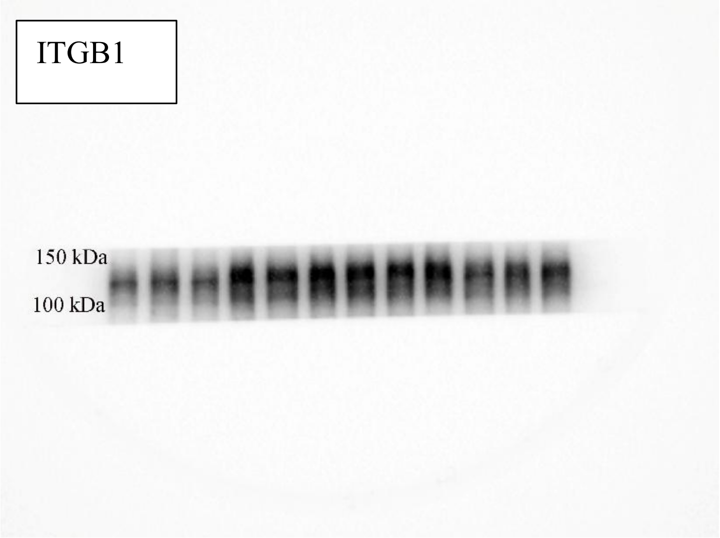
ITGB1
150 kDa
100 kDa

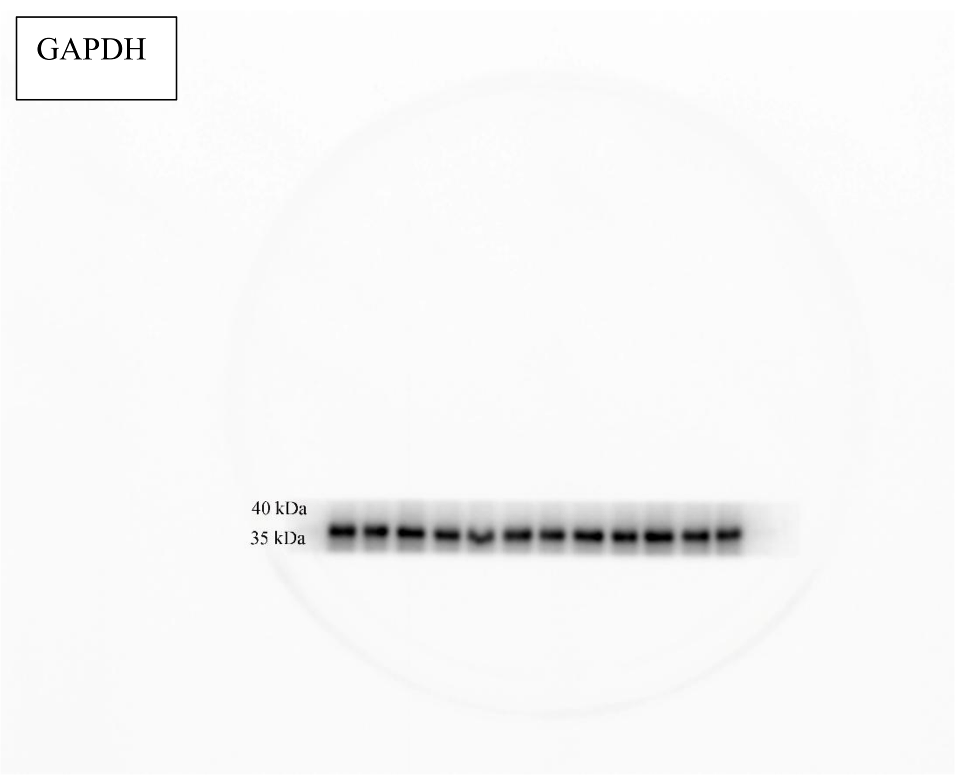
GAPDH
40 kDa
35 kDa

Fig.7e

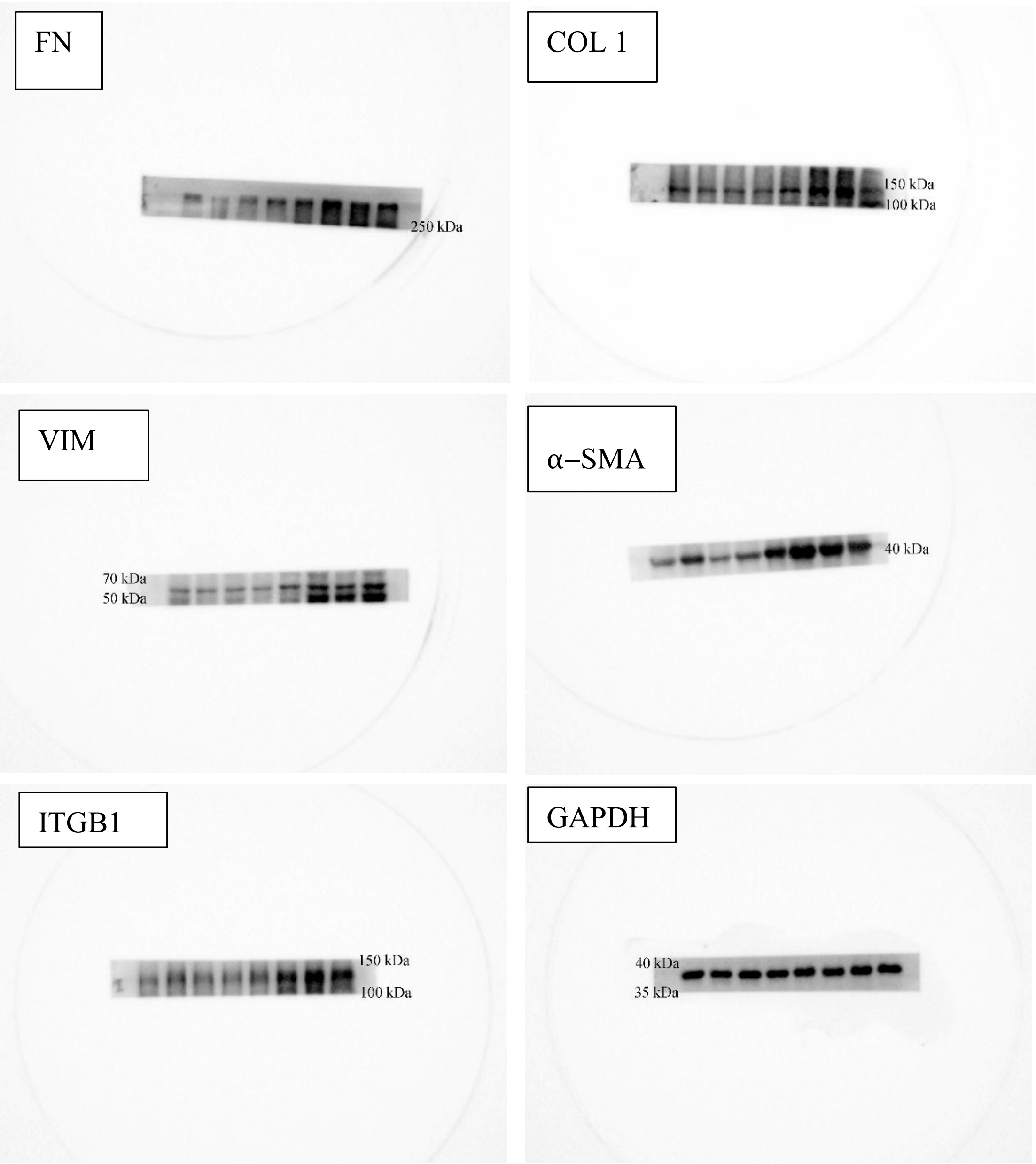
FN
250 kDa
COL 1
150 kDa
100 kDa
VIM
70 kDa
50 kDa
α−SMA
40 kDa
ITGB1
150 kDa
100 kDa
GAPDH
40 kDa
35 kDa

Fig.7f

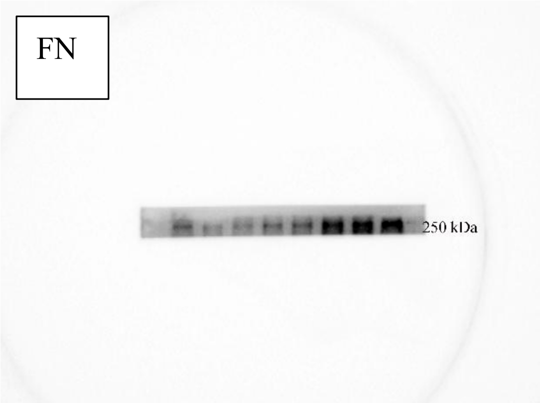
FN
250 kDa

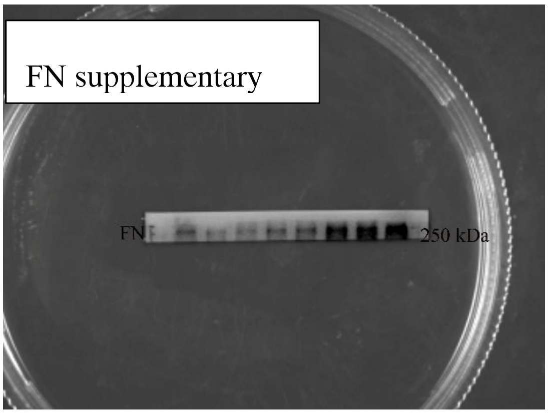
FN supplementary
FN
250 kDa

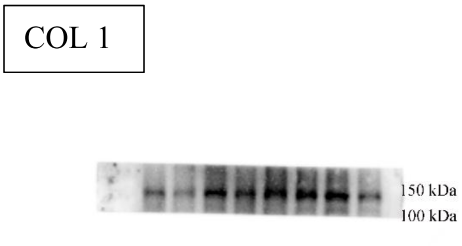
COL 1
150 kDa
100 kDa

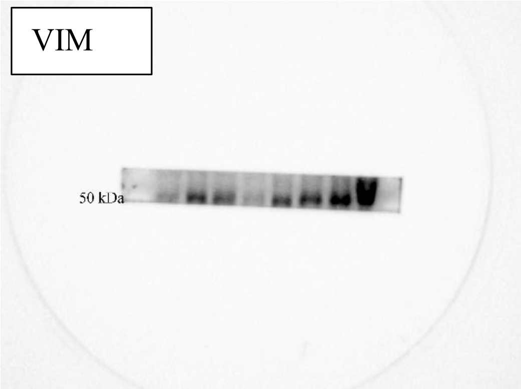
VIM
50 kDa

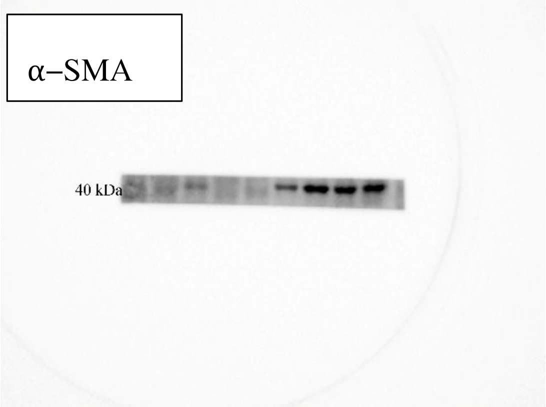
α−SMA
40 kDa

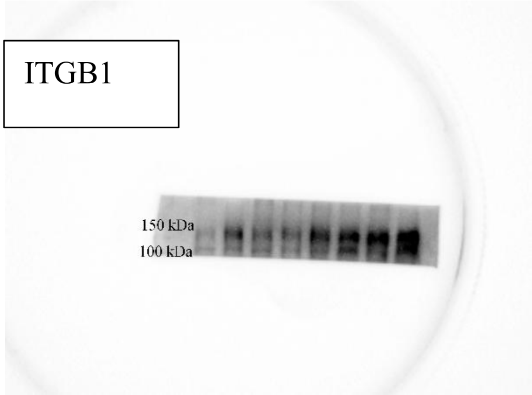
ITGB1
150 kDa
100 kDa

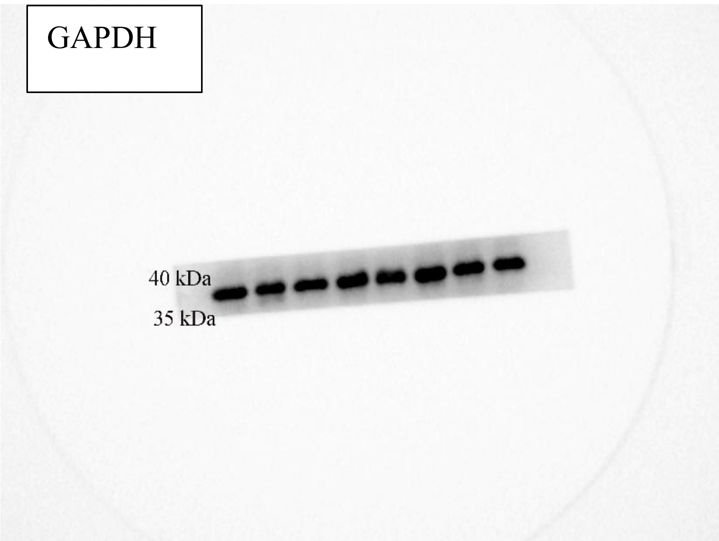
GAPDH
40 kDa
35 kDa
