# Supplementary figures and images for: Knockdown of lncRNA MALAT1 attenuates renal interstitial fibrosis through miR-124-3p/ITGB1 axis
Source: Sci Rep. 2023 Oct 23;13:18076. doi: 10.1038/s41598-023-45188-y (PMC10593763; doi:10.1038/s41598-023-45188-y)

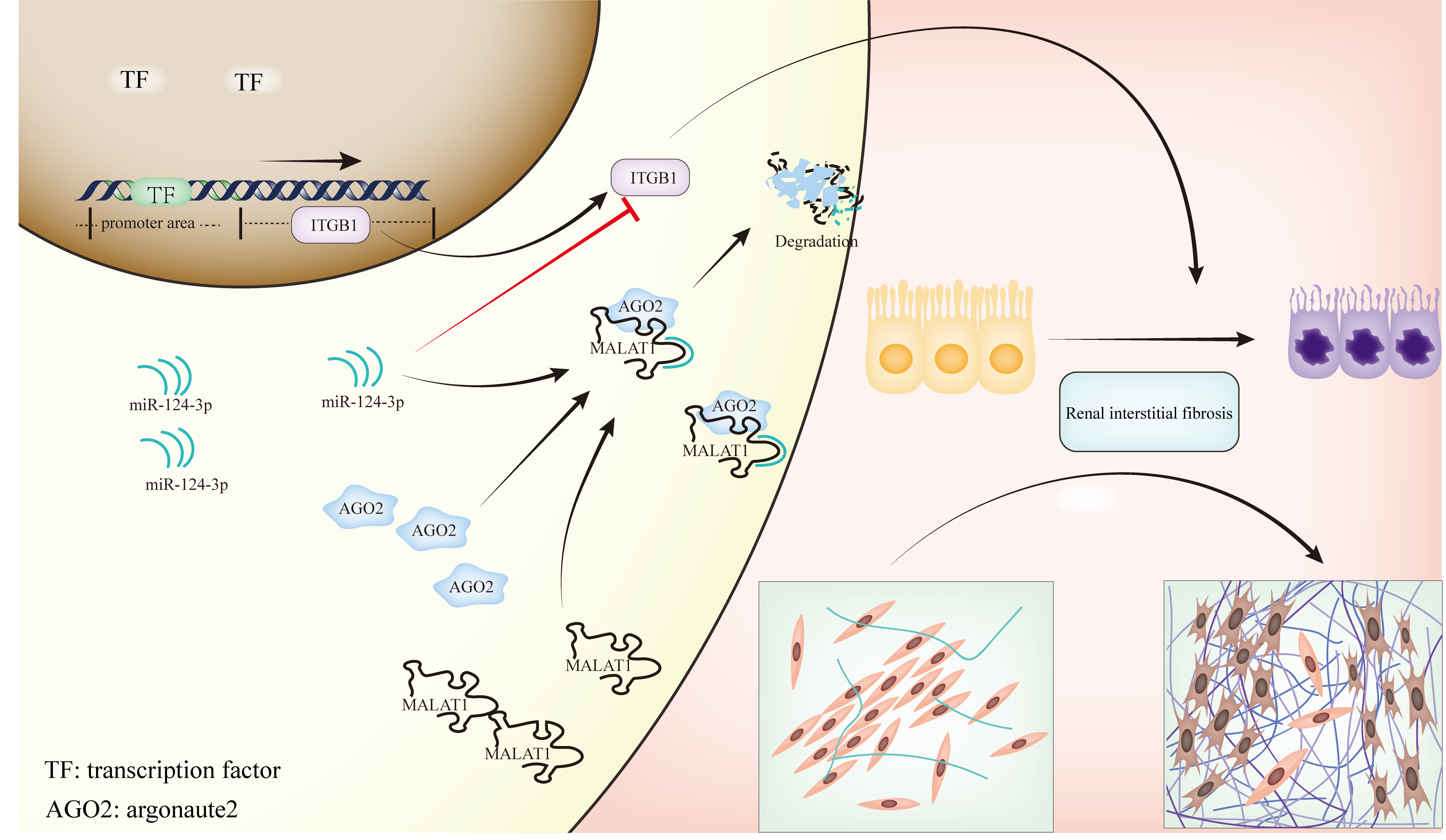

Supplement: Supplementary file 2 — Supplementary Figure 1. [file 41598_2023_45188_MOESM2_ESM.tif]
