# Supplementary material for: Knockdown of lncRNA MALAT1 attenuates renal interstitial fibrosis through miR-124-3p/ITGB1 axis
Source: Sci Rep. 2023 Oct 23;13:18076. doi: 10.1038/s41598-023-45188-y (PMC10593763; doi:10.1038/s41598-023-45188-y)
Supplement: Supplementary file 4 — Supplementary Legends. [file 41598_2023_45188_MOESM4_ESM.docx]

Supplementary Fig.1 Under disease conditions, lncRNA MALAT1 expression was up-regulated in *vivo* and in *vitro.* And lncRNA MALAT1 competitively bind to miR-124-3p using Ago2 containing antibody components and then regulate [ITGB1](https://pubmed.ncbi.nlm.nih.gov/31433301/) expression, thus leading to the promotion of RIF in *vitro* and in *vivo*.

Supplementary Fig.2 **a** WB showed that the expression changes of ITGB1 and fibrosis-related markers (E-ca, N-ca VIM, α-SMA) in HK2 cells under TGF-β1 concentration gradient (from 3 experiments). **b** WB was used to determine the expression changes of ITGB1 and fibrosis-related markers (FN, COL1, VIM, α-SMA) in NRK-49F cells under TGF-β1 concentration gradient (from 3 experiments). *：p＜0.05;**：p＜0.01;***：p＜0.001; ****：p＜0.0001(compare with 0 ng/ml); #：p＜0.05; ##：p＜0.01; ###：p＜0.001; ####：p＜0.0001 (compare with 2.5 ng/ml); &：p＜0.05; &&：p＜0.01(compare with 5 ng/ml).
