# Supplementary material for: Knockdown of lncRNA MALAT1 attenuates renal interstitial fibrosis through miR-124-3p/ITGB1 axis
Source: Sci Rep. 2023 Oct 23;13:18076. doi: 10.1038/s41598-023-45188-y (PMC10593763; doi:10.1038/s41598-023-45188-y)
Supplement: Supplementary file 5 — Supplementary Table 1. [file 41598_2023_45188_MOESM5_ESM.doc]

**the PCR primer sequences information**

| Gene | Forward primer 5**,**-3**,** | Reverse primer5**,**-3**,** | | |
| --- | --- | --- | --- | --- |
| hsa-lncRNA MALAT1 | AAAGCAAGGTCTCCCCACAAG | GGTCTGTGCTAGATCAAAAGGCA | | |
| hsa-ITGB1 | GTTCAGTTTGCTGTGTGTTTGC | ATCCTCTGGCTTGAGCTTCTCT | | |
| hsa-GAPDH | GGGGCTCTCCAGAACATC | TGACACGTTGGCAGTGG | | |
| hsa-miR-124-3p | CGTAAGGCACGCGGTGAA | AGTGCAGGGTCCGAGGTATT | | |
| hsa-miR-124-3p(RT-primer) | GTCGTATCCAGTGCAGGGTCCGAGGTATTCGCACTGGATACGACTTGGCA | | | |
| U6 | GGAACGATACAGAGAAGATTAGC | | TGGAACGCTTCACGAATTTGCG | |
| mmu-lncRNA Malat1 | ACCTCCCAGTTTTGTAAGACG | | CCAATTACCTCCCCTACACA | |
| mmu-Itgb1 | AGCAACAATTCACCCACAG | | TTCGAGACAGAGCAAGCA | |
| mmu-Gapdh | TGTTTCCTCGTCCCGTAGA | | ATCTCCACTTTGCCACTGC | |
| mmu-mir-124-3p | AGTGCAGGGTCCGAGGTATT | | GCGTAAGGCACGCGGTG | |
| mmu-mir-124-3p(RT-primer) | GTCGTATCCAGTGCAGGGTCCGAGGTATTCGCACTGGATACGACGGCATT | | | |
| rat-lncRNA Malat1 | TGGGAATGGTCTTAACAGGGAGGAG | | | AACAGCATAGCAGTACACGCCTTC |
| rat-Itgb1 | CGTGCGGAAGACAAGTG | | | CTCACAATGGCACACAGG |
| rat-Gapdh | ATGGCTACAGCAACAGGGT | | | TTATGGGGTCTGGGATGG |
| rat-mir-124-3p | AGTGCAGGGTCCGAGGTATT | | | GCGTAAGGCACGCGGTG |
| rat-mir-124-3p(RT primer) | GTCGTATCCAGTGCAGGGTCCGAGGTATTCGCACTGGATACGACGGCATT | | | |
